# Advancements in research on the precise eradication of cancer cells through nanophotocatalytic technology

**DOI:** 10.3389/fonc.2025.1523444

**Published:** 2025-04-01

**Authors:** Changyang Yao, Chensong Zhang, Dongwei Fan, Xuanhe Li, Shaofa Zhang, Daoxin Liu

**Affiliations:** ^1^ Department of General Surgery, Fengyang County People’s Hospital, Chuzhou, China; ^2^ Department of Surgical Oncology Surgery (General Ward), The First Affiliated Hospital of Bengbu Medical College, Bengbu, China; ^3^ Department of General Surgery, Affiliated Hospital of West Anhui Health Vocational College, Lu’an, Anhui, China

**Keywords:** cancer, high conduction band, high valence band, composites, S-scheme

## Abstract

The rapid development of nanotechnology has significantly advanced the application of nanophotocatalysis in the medical field, particularly for cancer therapy. Traditional cancer treatments, such as chemotherapy and radiotherapy, often cause severe side effects, including damage to healthy tissues and the development of drug resistance. In contrast, nanophotocatalytic therapy offers a promising approach by utilizing nanomaterials that generate reactive oxygen species (ROS) under light activation, allowing for precise tumor targeting and minimizing collateral damage to surrounding tissues. This review systematically explores the latest advancements in highly efficient nanophotocatalysts for cancer treatment, focusing on their toxicological profiles, underlying mechanisms for cancer cell eradication, and potential for clinical application. Recent research shows that nanophotocatalysts, such as TiO_2_, In_2_O_3_, and g–C_3_N_4_ composites, along with photocatalysts with high conduction band or high valence band positions, generate ROS under light irradiation, which induces oxidative stress and leads to cancer cell apoptosis or necrosis. These ROS cause cellular damage by interacting with key biological molecules such as DNA, proteins, and lipids, triggering a cascade of biochemical reactions that ultimately result in cancer cell death. Furthermore, strategies such as S–scheme heterojunctions and oxygen vacancies (OVs) have been incorporated to enhance charge separation efficiency and light absorption, resulting in increased ROS generation, which improves photocatalytic performance for cancer cell targeting. Notably, these photocatalysts exhibit low toxicity to healthy cells, making them a safe and effective treatment modality. The review also discusses the challenges associated with photocatalytic cancer therapy, including limitations in light penetration and the need for improved biocompatibility. The findings suggest that nanophotocatalytic technology holds significant potential for precision cancer therapy, paving the way for safer and more effective treatment strategies.

## Highlights

- Nanophotocatalytic therapy precisely targets cancer cells through regulated reactive oxygen species (ROS).
- S-scheme heterojunctions and oxygen vacancies improve light absorption and ROS generation in nanophotocatalysts.
- High efficacy and low toxicity position nanophotocatalytic technology as a promising cancer treatment option.

## 1 Introduction

The rapid progression of nanotechnology has positioned nanophotocatalysis at the forefront of contemporary scientific inquiry, owing to its extensive applications across energy conversion, environmental remediation, and biomedical sciences (1–8). Nanophotocatalysts (Nanophotocatalysts are photocatalysts in which the size of the particles constituting the photocatalyst reaches the nanometer order of magnitude (10^–9^ m). When the particle size reaches the nanometer level, it reveals magnetic, optical, acoustic, thermal, electrical, and superconducting properties that are significantly different from those of macroscopic objects, and thus has unique photophysical properties and high photocatalytic activity), typically ranging from one to several hundred nanometers in size, exhibit unique optical, chemical, and electronic properties that enable them to harness light energy to generate the electron–hole pairs, thereby initiating a variety of redox reactions (9–16). Over the past few decades, significant advancements have been made in utilizing nanophotocatalysis for pollutant degradation, water purification, and renewable energy generation (17–26). However, the efficacy of single–component nanophotocatalysts has been hindered by limitations such as suboptimal photocatalytic efficiency, poor stability, and low charge carrier separation efficiency (27–38). To surmount these challenges, innovative strategies like the incorporation of S–scheme heterojunctions (An interfacial region formed by two or more different materials (usually semiconductors, but can also be conductors or insulators). These materials, when in contact, form a heterojunction because they have different energy band structures, electron mobility, or chemical properties) and the introduction of oxygen vacancies (OVs) have been employed to enhance photocatalytic performance by improving charge separation and augmenting light absorption, ultimately leading to elevated generation of reactive oxygen species (ROS) (A general term for oxygen–containing free radicals and free radical–prone peroxides associated with oxygen metabolism in living organisms. Include superoxide radical anion (·O_2_^–^), other oxygen radicals, non-radical derivatives of O_2_, ozone (O_3_), singlet oxygen (^1^O_2_), hydroxyl radicals (·OH), and other substances (39–58).

The interdisciplinary nature of nanophotocatalysis has made it a focal point of modern scientific research, intersecting fields such as physics, chemistry, materials science, biomedicine, and environmental science. Nanophotocatalysis is a cutting–edge technology based on the generation of catalytic reactions by nanomaterials under light. The core of this technology is to utilize the photocatalytic properties of nanoscale semiconducting materials (e.g., titanium dioxide, zinc oxide, etc.) to generate ROS or other highly reactive substances under light, thus triggering chemical reactions. This technology was initially widely used in environmental fields, such as air purification, water treatment, and pollutant degradation, and has attracted much attention due to its high efficiency and environmentally friendly properties (59–62). In recent years, with the cross development of nanotechnology and biomedicine, nanophotocatalytic technology has been gradually introduced into the biomedical field, showing great potential in cancer treatment. The basic principle is to induce apoptosis or necrosis of cancer cells by generating ROS, such as **·**OH and superoxide anions, through the photosensitizing properties of nanomaterials, which drive redox reactions under light irradiation at specific wavelengths (63–71). Recently, a drug–free tumor treatment concept, nanophotocatalysis, was proposed by Zhao and colleagues. A Z–type SnS_1.68_–WO_2.41_ nanocatalyst was developed to achieve the generation of near–infrared photocatalytic oxidized holes and hydrogen molecules, and to achieve combined hole/hydrogen treatment of tumors through a drug–free treatment strategy, exemplifying that nanophotocatalysis plays a key role. SnS_1.68_–WO_2.41_ nanocatalysts oxidized/consumed glutathione (GSH) overexpressed in tumors via cavities under near–infrared irradiation and simultaneously generated hydrogen molecules in a durable and controllable manner. The generated hydrogen molecules and consumed glutathione inhibited cancer cell energy and disrupted intratumoral redox balance, respectively, thereby synergistically damaging DNA and inducing tumor cell apoptosis. The results showed that the SnS_1.68_–WO_2.41_ nanocatalyst could effectively kill cancer cells and inhibit tumor growth after 22 days under NIR irradiation (72). In contrast, conventional cancer treatments—including surgery, radiotherapy, and chemotherapy—are often accompanied by severe side effects, such as damage to healthy tissues, systemic toxicity, and the emergence of drug resistance. Nanophotocatalysis, however, offers a more refined and precise therapeutic modality (73–81). By generating ROS through photocatalytic processes, tumor cells can be selectively targeted and eradicated without harming surrounding healthy tissues, thereby mitigating the adverse effects associated with traditional treatments (35, 82–89).

In the biomedical domain, particularly in cancer therapy, nanophotocatalysts have demonstrated immense potential. Materials such as TiO_2_, CeO_2_, and Fe_3_O_4_ not only efficiently produce ROS under ultraviolet or visible light irradiation but also modulate the tumor microenvironment to selectively eliminate malignant cells (90–101). These nanomaterials have been extensively employed in antibacterial, antiviral, and disinfection applications, with TiO_2_ being notably utilized in the development of photocatalytic disinfectants due to its potent oxidative properties under UV light (102–113). In the realm of photocatalytic cancer cell targeting, researchers like Divinah Manoharan and Ankush Sharma have engineered photocatalytic nanoparticles (CNPs) that generate ROS under specific wavelengths of light, facilitating targeted destruction of tumor cells while sparing normal tissues (114, 115).

This review delves into the advancements of photocatalysis in cancer treatment, with a particular emphasis on the design and development of highly efficient nanophotocatalysts, the underlying mechanisms of photocatalytic reactions, the strategies for cancer cell eradication, and their potential clinical applications. By integrating approaches such as S–scheme heterojunctions, engineering of OVs, and other synergistic mechanisms, the efficiency of light absorption and charge carrier separation in photocatalysts has been significantly enhanced. These enhancements lead to increased ROS production and precise targeting of cancer cells. This emergent technology showcases remarkable advantages in oncological treatments, offering superior photocatalytic performance and safety compared to conventional methodologies, while also exhibiting low toxicity and absence of drug resistance. Furthermore, this review addresses the challenges confronting photocatalytic cancer therapy and outlines future research directions and trends, providing valuable insights and guidance for advancing the field.

## 2 Nanophotocatalysts and quenching mechanism

Cancer, an intricate and multifactorial disease characterized by uncontrolled cellular proliferation and metastasis, arises from a confluence of endogenous and exogenous factors such as genetic mutations, hormonal imbalances, immune dysregulation, exposure to carcinogens, radiation, and oncogenic pathogens (28–35). Conventional therapies—including surgery, radiotherapy, and chemotherapy—while partially effective, are often accompanied by severe adverse effects, notably damage to healthy tissues and the development of multidrug resistance (36–40). In contrast, nanophotocatalytic therapy has emerged as a precise and minimally invasive modality that utilizes nanophotocatalysts activated by specific wavelengths of light to generate ROS, which selectively disrupt redox homeostasis in cancer cells, inducing apoptosis or necrosis while sparing normal tissues (41–45). The tunable physicochemical properties of these nanophotocatalysts enable personalized treatment strategies, aligning with the principles of precision medicine. Recent advancements in this field have demonstrated unprecedented therapeutic potential, propelling nanophotocatalytic therapy toward clinical application and heralding a paradigm shift in oncological treatment (36–38). Future research is expected to focus on optimizing photocatalytic efficiency, enhancing biocompatibility, and elucidating the molecular mechanisms underlying cancer cell quenching, positioning this technology as a formidable contender in next–generation cancer therapeutics.

### 2.1 Highly efficient nanophotocatalysts

Nanophotocatalysts are a class of nanomaterials that drive chemical reactions through light activation, typically ranging in size from one to several hundred nanometers. These catalysts function by absorbing photons, exciting internal electrons to higher energy states, and subsequently generating electron–hole pairs. These electron–hole pairs can actively participate in chemical reactions, facilitating the transformation of various reactants regardless of the reactants’ inherent photochemical activity (39–45). Due to their exceptional photoelectric properties, nanophotocatalysts have demonstrated remarkable applicability across various fields. Their applications span from water splitting for hydrogen production and pollutant degradation to photonic energy conversion, catalysis in organic synthesis, and precise medical interventions such as the elimination of cancer cells (46–54). By optimizing reaction conditions, these nanomaterials significantly enhance reaction efficiency, showing tremendous potential in energy production, environmental remediation, and biomedicine. Nanophotocatalytic technology not only fosters advancements in cutting–edge fields but also provides innovative solutions for green and sustainable technologies, illustrating expansive research and application prospects.

#### 2.1.1 TiO_2_/WO_3_ composites

TiO_2_/WO_3_ composites have demonstrated exceptional performance in the field of photocatalysis, particularly under visible light irradiation, where they effectively generate photoexcited electrons that interact with pollutants, rapidly degrading various contaminants and thereby mitigating the environmental threats posed by harmful substances (46–50). Heavy metal ions, with their high electron affinity, serve as ideal electron acceptors, enabling photoexcited electrons in TiO_2_/WO_3_ composites to directly participate in the reduction of heavy metals. This significantly reduces their potential harm to both the environment and human health. Although the presence of oxygen molecules may interfere with the storage and transfer of photoexcited electrons, this effect is not entirely negative. In fact, the reaction between photoexcited electrons and oxygen molecules generates ROS, including **·**OH and **·**O_2_^–^, which exhibit potent biological activity. These ROS can induce apoptosis in cancer cells and possess antiviral properties, opening up new avenues for the application of photocatalytic technology in medicine, particularly in cancer treatment and public health. Therefore, TiO_2_/WO_3_ composites not only excel in environmental pollution control but also present broad potential in the biomedical field (47–49).

The photocatalytic efficiency of TiO_2_/WO_3_ nanocomposites can be further enhanced by forming heterojunction structures between TiO_2_ and WO_3_ with OVs. For example, Li et al. confirmed the presence of OVs in WO_3_ using electron paramagnetic resonance (EPR) spectroscopy and, through theoretical calculations and EPR experiments, verified the S–scheme heterojunction structure of TiO_2_/WO_3_ nanocomposites (46). OVs effectively trap photoexcited electrons, reducing electron–hole recombination and accelerating charge separation and transfer, thereby enhancing the efficiency of photocatalytic reactions (47–49). Moreover, the presence of OVs increases the number of surface active sites, further strengthening interactions with reactants and boosting photocatalytic activity (50). The S–scheme heterojunction structure efficiently separates photoexcited electrons and holes, optimizing the electron–hole recombination process in photocatalysis and enhancing the generation of ROS (51, 52). These ROS exhibit strong oxidative capabilities, disrupting the molecular structures of cancer cells, including DNA, proteins, and lipids, ultimately leading to the loss of cancer cell function and cell death. Additionally, ROS can trigger pyroptosis, a form of cell death distinct from apoptosis, which releases immune–related factors that further stimulate immune responses to effectively eliminate cancer cells (53–58, 63–65). Furthermore, studies have shown that TiO_2_/WO_3_ nanocomposites exhibit significant bactericidal properties, capable of effectively killing E. coli within 6 hours under UV irradiation by disrupting bacterial membrane lipids through ROS (98, 99). This ROS–based disinfection mechanism offers a novel and effective solution for public health and sterilization applications.

#### 2.1.2 In_2_O_3_/WO_3_ composites

In_2_O_3_/WO_3_ composites, with their OVs and S–scheme heterojunction structures, have demonstrated excellent performance in photocatalytic degradation and mineralization processes. The S–scheme heterojunction optimizes the pathways for separating electrons and holes, significantly enhancing the separation efficiency of photogenerated charge carriers and reducing recombination rates. This, in turn, dramatically improves the photocatalytic efficiency. The photocatalytic activity of In_2_O_3_/WO_3_ is greatly influenced by various physical and chemical properties, including morphology, electronic structure, crystallinity, and surface exposure of crystals. By incorporating OVs and forming an S–scheme heterojunction, In_2_O_3_/WO_3_ composites have shown superior performance under complex reaction conditions, particularly excelling in the photocatalytic degradation of organic pollutants (66, 67).

**Figure 1:**
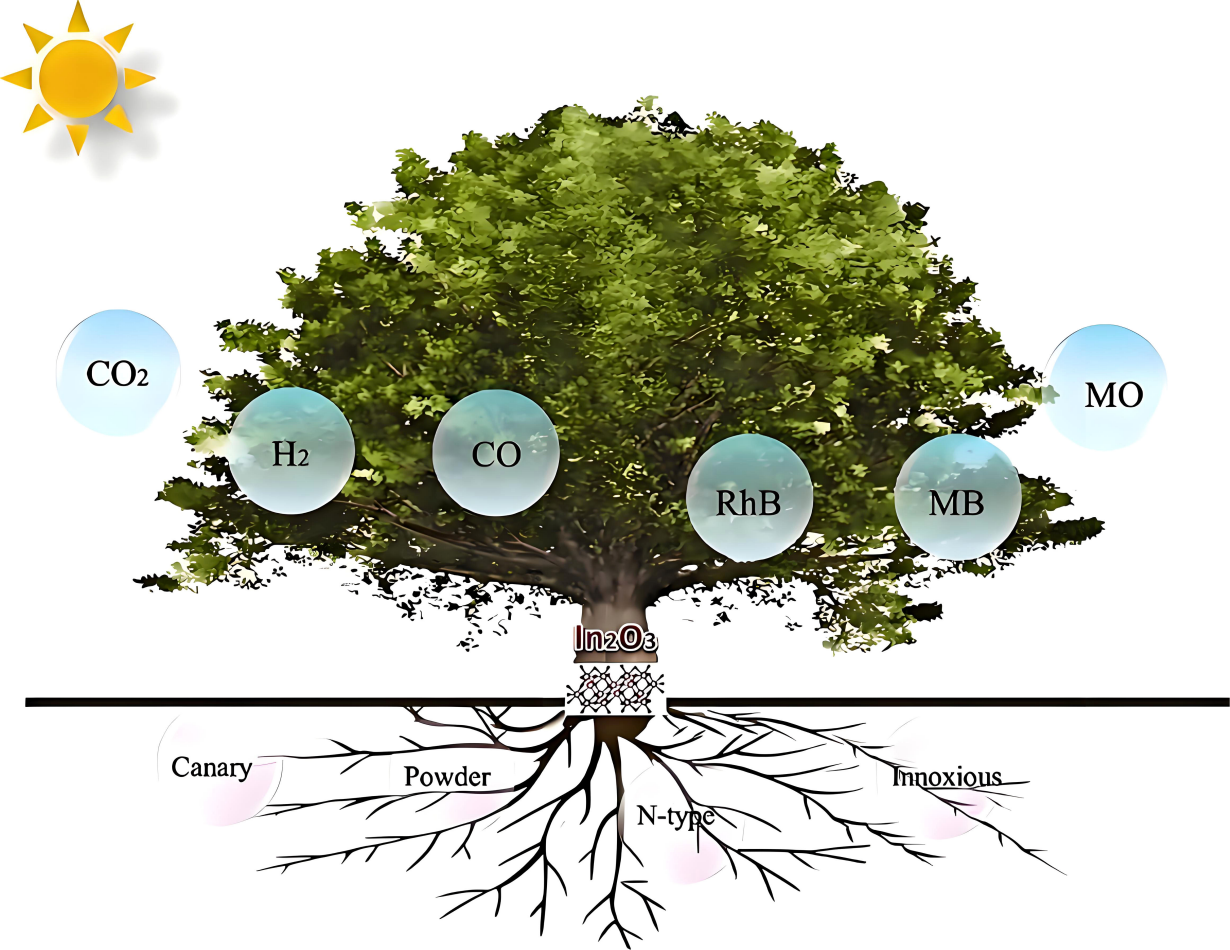
Illustrates the application of In_2_O_3_ in photocatalytic hydrogen production, carbon dioxide conversion, and pollutant degradation (80, 81).

Traditional photocatalysts like titanium dioxide (TiO_2_) and zinc oxide (ZnO) have wide band gaps, which limit their absorption to ultraviolet light, thereby restricting their application under visible light (68, 69). To overcome this limitation, researchers have focused on developing alternative semiconductor materials such as In_2_S_3_, CdS, and In_2_O_3_, aiming to enhance visible light absorption and overall photocatalytic efficiency (70, 71). The unique nanostructure of In_2_O_3_ not only exhibits excellent electrical and optical properties but also holds great potential in various applications, including photocatalytic hydrogen production, CO_2_ conversion, and organic pollutant degradation (shown in **Figure 1**). As a non–toxic n–type semiconductor, In_2_O_3_ is highly adaptable to structural design and doping modifications, making it an ideal candidate for developing new photocatalysts. However, the high recombination rate of photogenerated charge carriers and the inefficient utilization of photonic energy in single–component In_2_O_3_ limit its photocatalytic efficiency. Li and colleagues successfully addressed this issue by combining oxygen–deficient W_18_O_49_ with In_2_O_3_, forming an S–scheme heterojunction structure that significantly improved light utilization efficiency and reduced electron–hole recombination, thus enhancing overall photocatalytic performance (77–82).

Furthermore, Zn–doped In_2_O_3_ nanoparticles (NPs) have shown great promise in cancer therapy. Research by ZabnAllah and colleagues demonstrated that different molar ratios of Zn–doped In_2_O_3_ NPs (2.5%, 5%, and 7.5%) exhibited dose–dependent cytotoxic effects on MCF–7 breast cancer cells. Higher Zn doping levels lead to greater generation of ROS through photocatalysis, resulting in stronger cytotoxic effects. These ROS induce oxidative stress, damaging the DNA, proteins, and lipids of cancer cells, ultimately triggering apoptosis. Importantly, the study also revealed that In_2_O_3_ NPs exhibited good biocompatibility with normal human cells (HUVECs), selectively killing cancer cells without harming normal cells. These findings suggest that In_2_O_3_–based photocatalysts have the potential to serve as novel agents for photodynamic cancer therapy, further expanding the biomedical applications of nanophotocatalysis (35, 83–86).

#### 2.1.3 g–C_3_N_4_/WO_3_ composites

In the quest for highly efficient photocatalytic materials, researchers have increasingly focused on the development of composite semiconductors (85–89). Among these, composites based on graphitic carbon nitride (g–C_3_N_4_) and WO_3_ have emerged as frontrunners due to their exceptional optical and chemical properties (82, 90–95). g–C_3_N_4_ is renowned for its excellent chemical stability and moderate band gap, enabling efficient absorption of visible light in photocatalysis. However, despite its promising attributes, the overall photocatalytic efficiency of g–C_3_N_4_ remains suboptimal. Conversely, WO_3_, with its narrower band gap, demonstrates high photocatalytic activity but faces challenges when used independently due to low charge carrier separation efficiency and limited stability. Recent studies have shown that introducing OVs can significantly optimize the photocatalytic properties of WO_3_, greatly enhancing its potential in the field (7). It is noteworthy that while extensive research has been conducted on the application of g–C_3_N_4_/WO_3_ composites in environmental pollution control, investigations into their potential for cancer cell elimination are still in their infancy, presenting numerous research opportunities and significant scientific value for future innovative developments.

To overcome the inherent limitations of individual materials, recent research has increasingly focused on constructing composite materials by employing strategies such as S–scheme heterojunctions (100–103). This approach harnesses the strengths of both g–C_3_N_4_ and WO_3_, preserving their distinct band edge characteristics while significantly enhancing charge carrier separation efficiency and reducing electron–hole recombination. This synergistic effect not only improves the overall photocatalytic efficiency of the material but also enhances its performance under visible light by introducing OVs. As a result, the S–scheme heterojunction strategy substantially boosts the ROS generation capacity of g–C_3_N_4_/WO_3_ nanocomposites, rendering them highly promising for applications in nanophotocatalytic therapy, particularly in the elimination of cancer cells, and highlighting their significant potential for future development.

Hakimi–Tehrani et al. conducted research on the antibacterial potential of g–C_3_N_4_/WO_3_, demonstrating that this composite material exhibited strong inhibitory effects against Staphylococcus aureus and Escherichia coli. The antibacterial efficacy was particularly pronounced when the WO_3_ content reached 15% (104). Duan and colleagues further confirmed that W^6+^ ions generated by WO_3_ could attach to and penetrate bacterial cells, exerting bactericidal effects (105). Additionally, the ROS generated under light activation of g–C_3_N_4_/WO_3_ were found to compromise the structural integrity of bacterial cell membranes, serving as a key antibacterial mechanism (104). Zhang et al. explored the antiviral effects of g–C_3_N_4_/WO_3_/biochar composites on adenovirus, revealing that the material could inactivate viruses without requiring regeneration during continuous use. Transmission electron microscopy imaging displayed the rupture of viral envelopes and the leakage of genetic material, rendering the virus non–pathogenic (106, 107). These findings not only verify the potential of g–C_3_N_4_/WO_3_ in antibacterial and antiviral applications but also lay a solid foundation for its future use in cancer cell elimination.

#### 2.1.4 Indirect comparison of ROS efficiency

The above three types of nanophotocatalysts all have excellent photoelectric properties and have significant application prospects in various fields. As we mentioned above, the basic principle of nanophotocatalysts for tumor treatment is to rely on the photosensitivity of photocatalysts to induce apoptosis or necrosis of cancer cells by generating ROS such as **·**OH and **·**O_2_^–^ through redox reactions driven by light irradiation at specific wavelengths. In contrast, the integration of S–scheme heterojunctions, the introduction of OVs, and other synergistic mechanisms in nanophotocatalysts can significantly enhance the photon absorption and improve the charge–carrier separation efficiency, thereby increasing the generation of ROS. Existing research results have shown that ROS such as **·**OH and **·**O_2_^–^ generated by nanophotocatalysts have highly efficient degradation capabilities for organic pollutants such as organophosphorus pesticides and veterinary drugs. Therefore the efficiency of nanophotocatalysts to degrade organic pollutants to indirectly respond to the efficiency of ROS generation. For the quantum efficiency, in the photocatalytic process, hydrogen is mainly produced through the reaction of **·**O_2_^–^ with water or ethanol, and we can indirectly map the quantum efficiency of ROS through the apparent quantum efficiency of photocatalytic hydrogen production. The integration of S–scheme heterojunction and the introduction of OVs to enhance the degradation efficiency of organic pollutants and the apparent quantum efficiency of photocatalytic hydrogen production by different nanophotocatalysts were compared by reviewing the literature, and the results are shown in **Tables 1**, **2**.

**Table 1:** Comparison of degradation of organic pollutants by different nanophotocatalysts.

Nanophotocatalyst	ROS	Organic pollutant	Time (min)	Degradation Rate (%)	Reference
TiO_2_/WO_3_	**·**O_2_^−^,**·**OH	Triazophos	120	78.0	(46)
In_2_O_3_/WO_3_	**·**O_2_^−^,**·**OH	Triazophos	60	78.7	(82)
g–C_3_N_4_/WO_3_	**·**O_2_^−^,**·**OH	Triazophos	100	87.1	(116)
ZnO/WO_2.72_	**·**O_2_^−^,**·**OH	Triazophos	80	69.2	(62)
Al_6_Si_2_O_13_/WO_2.72_	**·**O_2_^−^,**·**OH	Triazophos	140	86.3	(61)
BiOCl–TiO_2_	**·**O_2_^−^,**·**OH	Norfloxacin	60	90.2	(117)

**Table 2:** Comparison of quantum efficiency of photocatalytic hydrogen production with different nanophotocatalysts. .

Nanophotocatalyst	ROS	Catalytic substrate	Apparent quantum efficiency (%)	Reference
Cd_0.5_Zn_0.5_S	**·**O_2_^−^,**·**OH	H_2_O	>89.0	(118)
Pt–PdS/CdS	**·**O_2_^−^,**·**OH	H_2_O	93.0	(119)
CoS_2_/Zn_3_In_2_S_6_	**·**O_2_^−^,**·**OH	H_2_O	66.2	(120)
Polyheptazine imide/Pt	**·**O_2_^−^,**·**OH	C_2_H_5_OH	73.0	(121)
Polyheptazine imide	**·**O_2_^−^,**·**OH	(CH_2_OH)_2_	62.3	(122)

### 2.2 Photocatalysts for cancer therapy

As current cancer treatment methods struggle with severe side effects and the challenge of incomplete cures, the scientific community is vigorously exploring novel therapeutic strategies that are faster, more thorough, highly targeted, and safer. Photocatalytic elimination therapy, an emerging approach in cancer treatment, has garnered significant attention from researchers and clinical practitioners due to its mechanism of utilizing photocatalysts under specific light irradiation to generate ROS that directly attack cancer cells. Compared to traditional chemotherapy and radiotherapy, nanophotocatalysts exhibit distinct advantages; these catalysts synergistically combine the excellent optical and physicochemical properties of inorganic materials with the targeted functionalities of biomolecules, thereby enhancing therapeutic efficacy. Moreover, photocatalytic therapy can incorporate multifunctional drug molecules, achieving a synergistic effect that enhances precision and safety in treatment (108, 109). For example, a supramolecular photocatalyst, Nano–SA–TCPP (nanoporphyrin metal–organic framework), was developed by Zhu and colleagues. In an animal model, cancer cells were injected into the right dorsal subcutaneous culture of mice when the tumor volume exceeded ∼100 mm^3^, and solid tumors were treated with light irradiation at a wavelength of 600–700 nm. Experimental results showed that solid tumors (100 cubic mm^3^) were eradicated in as little as 10 minutes and the survival rate of mice increased from 0% to 100% within 50 days after treatment (110). Li and his team used ultrathin copper–tetrathione (4–carboxyphenyl) porphyrin (Cu–TCPP) MOF nanosheets to inject tumor–bearing mice, and then photothermal and photocatalytic irradiation was performed with an 808 nm laser and a 660 nm laser. Laser for coordinated photothermal and photocatalytic treatment, which showed that the cancer cells showed malignant cell shrinkage, nuclear condensation and fragmentation, which improved the survival rate of mice. Chen and his team designed and synthesized a gadolinium–porphyrin–based polymer, which was injected into the tail vein of mice and irradiated with a 635 nm laser for 10 minutes, resulting in the killing of more than 90% of the cancer cells (113). **Tables 3**–**5** summarize in detail several commonly used photocatalysts for cancer therapy and their characteristics, accompanied by the performance of photocatalytic treatment of tumors in animal experiments, which provide important insights into the advancement of this promising therapeutic modality (111–115, 123–143).

**Table 3:** Classes of inorganic nanophotocatalysts for quenching cancer cells.

	Photocatalyst	Physically trigger	Characterization	Mechanism	Performance	References
1	Zinc Oxide Nanoparticles	/	High biocompatibility and low toxicity	ROS production leads to cell death	Effectively induces natural apoptosis of adenocarcinoma cells	(123, 124)
2	Bismuth–based nanoparticles and composites	X–ray	High X–ray attenuation coefficient and near–infrared (NIR) absorbance, excellent photothermal conversion efficiency and long cycle half–life	Inducing DNA breaks in cancer cells	Tumor volume was reduced by 30%	(125)
3	CeO_2_/CuO heterogeneous structure	808 nm/10 min	Excellent tumor targeting properties	Generates ROS to induce cancer cell death	14 days cancer cell death	(126)
4	Carbon–based nanocomposite	808 nm/10 min	Efficiently absorbs light energy and converts it into heat energy	Chemotherapy/photothermal/photodynamic therapy synergistic modalities to generate ROS	Cancer cell activity decreased by 87.35% and died after 14 days	(127, 128)
5	One–dimensional TiO_2_ whiskers	Ultraviolet ray	Excellent photocatalytic activity and biocompatibility	Synergistic effect of photocatalytic TiO_2_ generation of ROS in combination with Zoerythromycin	Photocatalysis greatly enhances the mortality of cancer cells	(129)
6	TiO_2_ NPs	PH	Low–toxicity and stable	Delivery of doxorubicin induces cancer cell death	Significant programmed cell death	(130, 131)
7	Ag and Ag_2_O nanoparticles	1064 nm/10 min	No damage to other organs or cells	Photothermal effect synergy	It’s virtually eliminated in four days and won’t come back.	(132)

**Table 4:** Classes of organic nanophotocatalysts for quenching cancer cells.

	Photocatalyst	Physically trigger	Characterization	Mechanism	Performance	References
1	Supramolecular porphyrin photocatalysts	600–700 nm/10 min	Biocompatible, non–toxic, easy to metabolize	Photogenerated holes and electrons generate **·**OH and **·**O_2_^–^	Elimination of 100 mm^3^ solid tumor in 10 min	(125)
2	Nanogels	/	Rapid and controlled drug release in the tumor microenvironment	Chemotherapeutic paclitaxel (PTX) and immunotherapeutic agent interleukin–2 (IL–2)	Tumor inhibition rate of 74.7% within 14 days	(133)
3	TAF–(Triphenylamine (TPA) and hexylamine–substituted dibenzothiophene sulfone building blocks)	Near infrared light	Excellent biosafety, ultra–high cytotoxicity to hypoxic cells	Oxidative stress and bioreduction after photocatalysis	Significantly inhibits the growth of cancer cells	(134)
4	AlPCS4: aluminum(III) chloride phthalocyanine tetrasulfonate)	635 nm/0–20 min	Good cellular uptake efficiency, good biocompatibility and significant phototoxicity	Generation of single–linear oxygen species induces cancer cell death	The survival rate of cancer cells in the body drops dramatically.	(135, 136)
5	thienyl–substituted diketo pyrrolopyrrole (TDPP)	Xenon lamp	Excellent water solubility, biocompatibility and photostability	Cell death induced by single–linear oxygen species	Cancer cell viability reduced to 20%	(137)
6	4,6,4’–trimethylangelicin	Blue light	High antiproliferative activity	ROS burst cancer cells	Extremely effective	(138)
7	Multiple mitochondrial targeting motifs and ruthenium complexes (cHSA–PEO–TPP–Ru)	LED–light(470 nm/5 min)	Highly phototoxic, biodegradable	Generation of large amounts of unilinear oxygen to induce cancer cell death	Significantly enhanced phototoxicity of about 220–fold and phototoxicity to a wide range of cancer cells	(139)
8	Biomimetic poly(2–methacryloyloxyethyl phosphorylcholine)–b–poly(n–butyl methacrylate) (PMPC–b–PBMA) nanoparticles	Near infrared light(808 nm/1 min)	Good dispersion and remarkable stability	Photothermal effect	Over 80% of cancer cells are killed	(140, 141)
9	Benzene dithiophene–based polymers	LED/660 min	Strong absorption, high biocompatibility and superior stability	Phototherapy and photothermal therapy together	Most of the cancer cells are killed	(142, 143)

**Table 5:** Classes of hybrid nanophotocatalysts for quenching cancer cells.

	Photocatalyst	Physically trigger	Characterization	Mechanism	Performance	References
1	Nanoporphyrin metal–organic frameworks	650 nm/15 min	Produces abundant singlet oxygen with good photo–thermal conversion	Generates **·**O_2_^–^ to kill cancer cells	Kills 85% of cancer cells in 15 min	(111)
2	Copper–tetraketo(4–carboxyphenyl)porphyrin MOF nanosheets	808 nm/10 mim	Ultra–thin properties and good dispersion	Generates single–line oxygen to kill cancer cells	Tumor regression in 14 days	(112)
3	Gadolinium porphyrin supramolecular nanoparticles	635 nm/10 min	Good unilinear oxygen generation properties; excellent long–term colloidal stability, dispersibility and biocompatibility	Single–linear oxygen kills cancer cells	More than 90% of cancer cells killed in 10 min	(113)
4	Metal–Organic Framework/Titanium Dioxide Nanocomposite	983 nm/15 min	Good biocompatibility and good tumor cell killing properties	**·**O_2_^–^, **·**OH and ^1^O_2_ synergy	Severe destruction of cancer cells in 14 days	(114)
5	Manganese–iron oxide metal–organic framework nanocomplexes	660 nm/8 min	Regulation of tumor hypoxia and reducibility	^1^O_2_ and **·**O_2_^–^ synergy	Cancer cells within two weeks	(115)

### 2.3 Toxicological properties of photocatalysts

Extensive scientific research has elucidated the biological impacts of the highly efficient photocatalysts previously discussed. For example, studies have demonstrated that nano–TiO_2_ particles, once internalized by biological systems, can activate and induce interactions with alveolar macrophages, phagocytes, and microglial cells, leading to the generation of ROS (144). The production of ROS is closely linked to oxidative stress responses within cells, which can compromise membrane integrity and function, potentially triggering inflammation or cellular damage. Additionally, *in vivo* experiments and oral ingestion of nano–TiO_2_ have shown that these particles can enter the bloodstream, potentially affecting liver and kidney function and causing organ damage. Nano–TiO_2_ and its aggregates can also enter cells through interactions with surface receptors, and once internalized, they may exert mechanical stress on cell membranes, thereby affecting the stability and activity of membrane–associated receptors and ion channels (145, 146).

However, these findings regarding the toxicity of nano–TiO_2_ do not imply uncontrollable risks for humans or the environment. Recent studies have revealed that at lower concentrations, nano–TiO_2_ exhibits negligible toxicity (147, 148). With its excellent biocompatibility and superior drug delivery capabilities, nano–TiO_2_ has demonstrated significant potential in targeted cancer therapy and tumor treatment. By interacting with cancer cell membranes, nano–TiO_2_ effectively induces the production of ROS, such as **·**O_2_^–^ and **·**OH, disrupting cancer cell structures and enhancing the efficacy of cancer therapies (149–152). Moreover, nano–TiO_2_ is widely utilized in photothermal therapy (PTT), photodynamic therapy (PDT), and sonodynamic therapy (SDT), where it facilitates precise targeting and control via external stimuli, achieving targeted delivery and treatment of cancer cells (149, 153, 154). Given its low phototoxicity and high biocompatibility, nano–TiO_2_ holds great potential in phototherapy applications, demonstrating notable therapeutic effects in preclinical and clinical studies (130, 131).

Similar to nano–TiO_2_, nano–In_2_O_3_ exhibits excellent chemical stability and low toxicity. At ambient temperature and pressure, In_2_O_3_ is resistant to spontaneous decomposition, significantly reducing its toxicity risk during storage and application. Existing studies suggest that In_2_O_3_’s acute toxicity is relatively low, and short–term exposure to high doses inflicts minimal harm to biological organisms (155). Furthermore, reports indicate that workers exposed to indium over extended periods have shown no direct health abnormalities linked to indium exposure. Additionally, nano–In_2_O_3_, when combined with reduced graphene oxide (RGO), exhibits enhanced anticancer activity in colorectal and liver cancer cells while maintaining superior biocompatibility with normal cells (156). Meanwhile, g–C_3_N_4_, a non–metal semiconductor material composed of carbon and nitrogen, is generally considered to have low toxicity. In cellular experiments, low concentrations of g–C_3_N_4_ caused minimal morphological changes in cells, suggesting its low toxicity (157). Moreover, systemic administration and intratumoral injection of g–C_3_N_4_ demonstrated favorable biocompatibility, and when coupled with localized light treatment, it effectively reduced tumor size (158). Research has further categorized WO_3_ as a low–toxicity substance, with studies by Samaneh et al. confirming that WO_3_–NS does not exhibit significant toxicity even at higher concentrations (159).

In conclusion, nanocomposite materials integrating TiO_2_, In_2_O_3_, and g–C_3_N_4_ exhibit great potential in the field of photocatalytic cancer therapy. These materials not only enhance photocatalytic performance through the design of composites but also demonstrate excellent biocompatibility and low toxicity, offering promising prospects for future cancer therapies. Researchers have optimized the structures and functionalities of these composites, improving the precision and efficacy of targeted therapies, thereby laying a solid foundation for the practical application of photocatalytic treatments.

### 2.4 Long–term toxicity solutions for nanophotocatalysts

The long–term toxicity of nanophotocatalysts is a problem that stems mainly from their bioaccumulation, metabolic impairments, and the potential inflammatory responses that they trigger. These toxic effects may lead to cellular oxidative damage, genetic mutations and increased risk of chronic diseases. In the following, how to solve its long–term toxicity problem is systematically elaborated from the perspectives of inhibition of toxicity accumulation meter, metabolic regulation, and inflammation inhibition.

Discussed from the perspective of inhibiting toxicity accumulation, the accumulation of toxicity can be reduced by designing photocatalytic materials strained. The surface charge and hydrophilicity of nanoparticles significantly affect their distribution and accumulation in biological tissues. It has been shown that surface–coated polyethylene glycol (PEG) or silicon dioxide (SiO_2_) can form a spatial site barrier that reduces the interaction of nanoparticles with cell membranes. For example, Mano and his team surface–modified TiO_2_ nanoparticles with polyethylene glycol (PEG) to eliminate nanoparticle aggregation. The results showed that modifying TiO_2_ with PEG reduced its cytotoxicity and decreased the induction of stress–related genes (160). In addition, by modulating the size of the nanoparticles (>20 nm), the catalytic activity can be maintained while avoiding the systemic toxicity triggered by too small particles (<10 nm) through glomerular filtration or the blood–brain barrier. Park’s team investigated the effects of Ag nanoparticles of different sizes (20, 80, and 113 nm) on cells. Comparisons were made in *in vitro* assays for cytotoxicity, inflammation, genotoxicity and developmental toxicity. The 20 nm Ag particles were found to have the most pronounced effects on cellular metabolic activity and membrane damage. While larger size Ag nanoparticles had less effect (161).

Discussing from the perspective of metabolic regulation, degradable carrier design can be performed to promote metabolism. That is, the use of biodegradable materials (e.g., chitosan, polylactic acid) as carriers for nanocatalysts can realize the gradual degradation of the materials into non–toxic small molecules that can be excreted via the kidneys or the intestines after completing the catalytic task. In their review, Karlsson and team mentioned that biodegradable polymer nanocarriers hold great promise for enhancing the efficacy and safety of cancer treatments as a drug delivery vehicle. The properties of the polymers can be customized to ensure effective delivery of specific anticancer drugs from small molecule drugs to biologics. Biodegradable polymers can be safely degraded under physiological conditions and are engineered to respond to environmental and external triggers for spatially and temporally controlled delivery through engineering innovations (162).

Discussed from an inflammatory response perspective, the inflammatory response can be slowed by constructing heterojunctions and inhibiting inflammatory signaling pathway activation. The first way is to construct heterojunctions (S–scheme heterojunctions mentioned above) that optimize the efficiency of photogenerated electron–hole separation and reduce nonessential ROS overproduction. Wang and coworkers proposed a new reversible use of semiconductor heterojunctions to modulate ROS levels. The method integrates two metal–based ROS scavengers containing n–type CeO_2_ nanoparticles and n–type copper–doped diatom biosilica (Cu–DBs) to form typical n–n semiconductor heterojunctions (Ce/Cu–DBs). Unlike single ROS scavengers or ROS–generating agents that control ROS levels, Ce/Cu–DBs can rapidly eliminate ROS via a cascade catalytic reaction and readily switch to ROS generation via a near–infrared (NIR)–triggered photocatalytic effect. This NIR–mediated ROS modulation system provides a noninvasive strategy for the reversible control of ROS levels *in vitro* and *in vivo* to reduce the inflammatory response of the organism (163). The second approach is to inhibit inflammatory signaling pathway activation. Inflammatory responses triggered by nanoparticles are often mediated through the NF–κB or NLRP3 pathways. ZnO nanoparticles with surface–modified polydopamine (PDA) have been found to reduce pro–inflammatory factor release by inhibiting TLR4/MyD88 (Signaling pathway consisting of Toll–like receptor 4 (TLR4) and myeloid differentiation factor 88 (MyD88)) signaling. After green synthesizing ZnO nanoparticles using Aloe vera extract, Tavakoli’s team used a one–step direct method to surface–modify the nanoparticles with polydopamine (PDA). The results of the study confirmed that the synthesized polydopamine–coated zinc oxide (PDA@ZnO) nanoparticles possess good biocompatibility, have a minimal effect on the inflammatory response of the body, and are not only non–toxic to human cells, but also significantly promote cell survival (164).

### 2.5 Photocatalytic cancer cell quenching characteristics

In recent years, metal oxide materials such as ZnO, TiO_2_, CuO, SiO_2_, iron oxides (including Fe_2_O_3_ and Fe_3_O_4_), and CeO_2_ have garnered significant attention in biomedical applications, particularly in anticancer and antitumor treatments, due to their distinctive physicochemical properties, low production costs, biocompatibility, and potent cytotoxicity (165–167). In one study, Rasha A. and colleagues synthesized Ag–doped WO_3_ (3% Ag/WO_3_) photocatalysts, which substantially enhanced the photocatalytic efficacy against human cervical cancer cells (HeLa cells). Their results showed that under light irradiation at a concentration of 100 μg/mL for 20 minutes, 3% Ag/WO_3_ achieved a 90% elimination rate of HeLa cells, underscoring the role of Ag doping in significantly amplifying anticancer effects (168). Similarly, Gao et al. engineered CeO_2_/CuO heterostructures anchored on upconversion nanoparticles (UCNPs), modifying cancer cell membranes to enhance ROS generation. This enabled a synergistic effect between photocatalytic therapy and chemotherapy. *In vivo* mouse experiments demonstrated that 10 minutes of treatment with CeO_2_/CuO–UCNPs under 808 nm near–infrared light resulted in substantial tumor inhibition (100 mm^3^), with no recurrence observed after 14 days, highlighting the long–term therapeutic potential of this treatment (126).

Mohd Javed and colleagues conducted an investigation into the cytotoxicity of nano–ZnO on various cancer cell types, including human liver cancer (HepG2), human lung adenocarcinoma (A549), human bronchial epithelial cells (BEAS–2B), and rat astrocytes and hepatocytes. Their findings revealed that nano–ZnO effectively induced apoptosis in these cancer cells while sparing normal rat cells. This selective apoptotic induction is believed to be mediated through the tumor suppressor gene pathway, facilitated by ROS generation (169). Tian et al. further demonstrated that nano–ZnO disrupts intracellular Zn homeostasis, leading to lysosomal and mitochondrial damage and inducing ROS production, ultimately resulting in cancer cell death (124, 170). Collectively, these studies provide substantial evidence for the efficacy of photocatalytic technology in the elimination of cancer cells, while showcasing the unique advantages and promising potential of metal oxide–based photocatalysts in advancing cancer treatment strategies.

### 2.6 Mechanism of photocatalytic cancer treatment

The mechanism underlying the photocatalytic elimination of cancer cells primarily relies on the chemical reactions initiated by photocatalytic materials under specific light irradiation conditions. The efficiency of the photocatalytic activity of nanophotocatalysts is directly correlated with their ability to eliminate cancer cells. When the energy of photons equals or exceeds the bandgap of the semiconductor material, nanophotocatalysts generate electron–hole pairs under illumination. These electron–hole pairs undergo two key processes. The first and more favorable process involves photo–induced charges participating in redox reactions; holes oxidize H_2_O and OH^−^ to form **·**OH (see Equation 1), while electrons reduce O_2_ to generate ROS, such as **·**O_2_^–^ (see Equation 2). These ROS induce oxidative stress within the cellular system, which subsequently triggers apoptosis or necrosis in the cells. In contrast, the less desirable second process involves the radiative or non–radiative recombination of electron–hole pairs (see Equation 3), rather than their participation in redox reactions, thereby diminishing the photocatalytic efficiency and weakening the cancer cell elimination capability (38, 72, 168, 171, 172).

**Figure 2:**
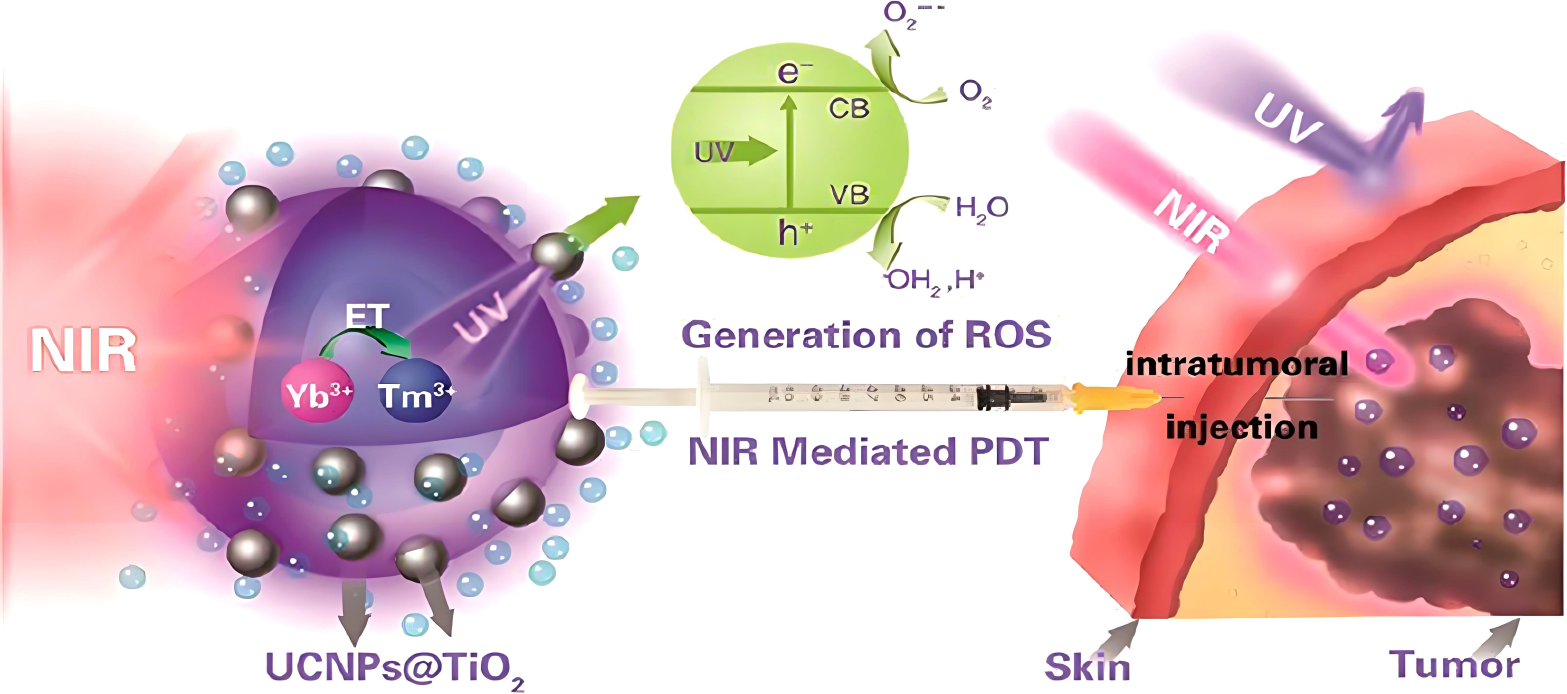
Tumor therapy facilitated by nano–TiO_2_ (152).

**Figure 3:**
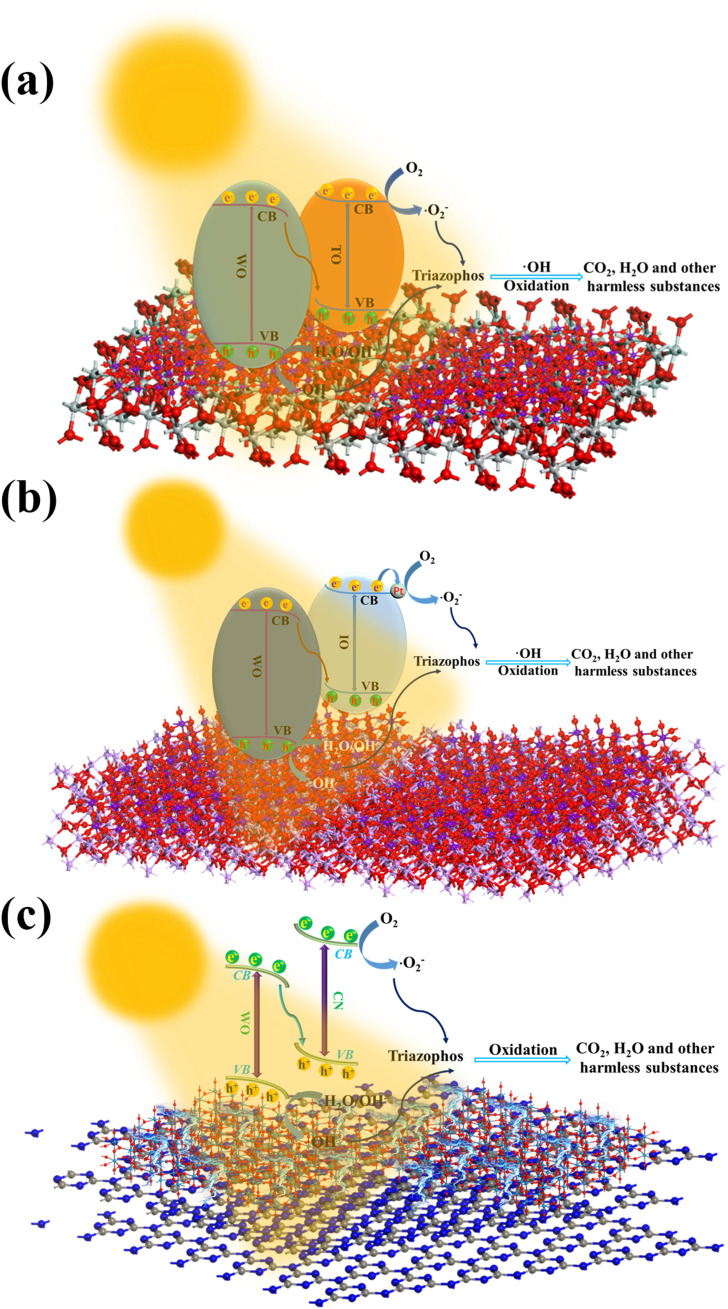
Mechanistic diagram illustrating the generation of ·OH and **·**O_2_^–^ by nanophotocatalysts (**a**: TiO_2_/WO_3_, **b**: In_2_O_3_/WO_3_, **c**: g–C_3_N_4_/WO_3_) (46, 82, 116).


To overcome the high recombination rate of the electron–hole pairs, researchers typically enhance photocatalytic performance by combining semiconductor photocatalysts with another suitable semiconductor to form heterojunctions or by doping them with noble metals (e.g., via Schottky junctions) to trap charges, thereby reducing recombination and improving photocatalytic efficiency. These modifications significantly elevate ROS production, thereby inducing more intense oxidative stress responses within cells, ultimately leading to apoptosis or necrosis. Such advancements substantially enhance the ROS generation capacity of nanophotocatalysts, amplifying their potential in cancer cell elimination applications (shown in **Figure 2**). **Figure 3** illustrates the mechanism by which the introduction of S–scheme heterojunctions and OVs elevates the levels of hydroxyl and **·**O_2_^–^. This strategy not only improves the degradation capacity of photocatalysts for organophosphorus pesticides but also provides robust scientific evidence and support for their application in the elimination of cancer cells.

(1) $${\text{h}}^{+}+{\text{H}}_{2}\text{O}/\text{O}{\text{H}}^{-}\to \cdot \text{OH}$$

(2) $${\text{e}}^{+}+{\text{O}}_{2}\to \cdot {\text{O}}_{2}{-}^{\;}$$

(3) $$\text{Photocatalyst}+\text{hv }\to \text{ Photocatalyst}({\text{h}}_{\text{vb}}{\;}^{+};{\text{e}}_{\text{cb}}{\;}^{-})\;\to \text{ recombination}$$

## 3 Applications of nanophotocatalysis in cancer cell ablation therapy

### 3.1 Upconversion nanoparticles in cancer cell ablation

Upconversion nanoparticles (UCNPs) are a unique class of nanomaterials characterized by their ability to absorb low–energy photons and emit high–energy photons—a phenomenon known as upconversion luminescence. Under near–infrared (NIR) light excitation, UCNPs emit high–energy visible light, which activates nearby photosensitizer (PS) molecules, resulting in the production of singlet oxygen or ROS that effectively kill cancer cells. Due to the superior tissue penetration of NIR light, UCNPs can facilitate photochemical reactions in deeper tissues compared to traditional visible or ultraviolet (UV) light exposure, thereby enhancing cancer treatment outcomes. In addition to serving as energy donors in photochemical processes, UCNPs can be utilized for NIR light–triggered drug release, imaging, and the activation of therapeutic molecules, achieving more precise cancer therapy (173, 174).

Wang and colleagues developed UCNP–Ce6 complexes by non–covalently binding Ce6 to a polyethylene–glycolated amphiphilic polymer–coated UCNP. After 30 minutes of exposure to 980 nm light at 0.5 W/cm^2^, the UCNP–Ce6 complexes successfully penetrated cancer cells and induced the death of 4T1 breast cancer cells in mice (175). Zhang’s team was the first to demonstrate the application of UCNPs in photodynamic therapy for breast cancer cells (MCF–7/AZ). Following 36 minutes of infrared irradiation, the breast cancer cells exhibited shrinkage and eventually died, showcasing the deep–tissue penetration and high specificity of UCNPs for targeting cancer cells (176, 177). Furthermore, Wang and his team utilized NaYF4 UCNPs co–doped with Yb^3+^ and Tm^3+^, which converted NIR photons into higher–energy photons, activating ZnO nanoparticles and generating a large amount of ROS, thereby significantly enhancing the anticancer effect (178). Gu and colleagues studied a system in which NIR laser radiation, through nonlinear optical interactions with tumor–targeting molecules, induced high–efficiency photocatalysis via single–photon absorption in ZnO, offering improved efficiency over conventional two–photon excitation (174). These studies provide strong evidence for the practical application of phototherapy in cancer treatment, demonstrating the vast potential of UCNPs as an emerging therapeutic modality.

### 3.2 TiO_2_ hybrid photocatalysis in cancer cell ablation

Under ultraviolet (UV) light excitation, TiO_2_ nanoparticles exhibit remarkable photocatalytic activity. However, UV light has significant limitations in penetrating biological tissues, with insufficient depth to effectively penetrate deep–seated cancer cells. This limitation hinders the efficacy of TiO_2_ nanoparticles in treating deep tumors *in vivo*. To overcome this drawback, researchers have developed hybrid systems by combining TiO_2_ with metals, metal oxides, or carbon nanomaterials to reduce its bandgap energy, thereby enhancing its photocatalytic activity under visible light and expanding the potential applications of photocatalysis in cancer treatment. For instance, incorporating SiO_2_ into TiO_2_ has been shown to improve its cytotoxicity against cancer cells. This combination broadens the light absorption spectrum and increases the photosensitivity to cancer cells (179). Such enhancements not only extend TiO_2_’s application in photodynamic therapy (PDT) but also offer promising therapeutic strategies for targeting cancer cells in deeper tissues.

Moreover, folic acid–conjugated SiO_2_–TiO_2_ nanoparticles, as a novel photosensitizer, have demonstrated superior active targeting capabilities in cancer treatment. Studies by Nurhidayatullaili et al. indicate that the addition of folic acid significantly inhibits cell proliferation and enhances the targeting of cancer cells. Under UV irradiation at various time points, folic acid–conjugated SiO_2_–TiO_2_ exhibited increased cytotoxicity against cancer cells. As the concentration of folic acid–conjugated SiO_2_–TiO_2_ nanocomposites increased, the survival rate of cancer cells notably decreased. In the presence of 12.5 µg/mL of folic acid–conjugated nanocomposites, the cancer cell survival rate dropped from 100% in the control group to 93%, 82%, and 78% at different time intervals, respectively. When the concentration of folic acid–conjugated SiO_2_–TiO_2_ increased to 100 µg/mL, the survival rate further decreased to 57% (179, 180). These findings not only highlight the potential of folic acid–conjugated SiO_2_–TiO_2_ in photocatalytic cancer therapy but also offer valuable insights for the future development and application of similar nanocomposites.

### 3.3 Novel photocatalytic ablation of cancer cells

Photocatalytic technology relies on generating a substantial amount of ROS to ablate cancer cells. However, this strategy is often hindered by the rapid recombination of the electron–hole pairs within the photocatalyst, limiting its efficacy. To address this limitation, researchers have developed a novel piezoelectric–assisted photocatalytic therapy that effectively enhances the separation of the electron–hole pairs at both bulk and interface levels, thereby triggering an intracellular ROS surge and inducing cancer cell apoptosis (181). Kang et al. employed calcination and liquid exfoliation techniques to synthesize heat–treated natural sphalerite nanosheets (NSH700 NSs), which exhibited remarkable piezoelectric photocatalytic effects. Under 660 nm laser irradiation for 10 minutes, combined with ultrasound stimulation, NSH700 NSs significantly reduced tumor volume (181). This enhanced photocatalytic performance is attributed to efficient charge separation and transfer mechanisms driven by a synergistic effect of polarized electric fields, band bending, and the unique heterojunction structure (182, 183). Compared to conventional photosensitizers, NSH700 NSs demonstrated superior photocatalytic activity, effectively disrupting the redox balance within cancer cells, ultimately leading to apoptosis. Cheng et al. further introduced a novel sonosensitizer, an oxygen–deficient piezoelectric nanocomposite (bismuth–doped oxygen–deficient barium titanate), which enhanced ROS production via sonodynamic therapy (SDT), significantly increasing the rate of tumor cell apoptosis (184–187).

As piezoelectric–assisted photocatalytic therapy continues to evolve, future research will delve deeper into its potential applications in cancer treatment. This innovative therapy not only facilitates direct tumor cell ablation through ROS generation but also synergizes with other mechanisms, such as thermoacoustic effects and enzyme catalysis, to further amplify therapeutic efficacy (188, 189). Looking ahead, piezoelectric photocatalytic materials are expected to achieve higher catalytic activity, improved biocompatibility, and reduced toxicity, offering safer and more effective options for cancer treatment. Additionally, this emerging technology lays a solid experimental foundation for broader biomedical applications, positioning piezoelectric–assisted photocatalytic therapy as a promising frontier in oncological treatment.

## 4 Challenges and prospects of photocatalytic cancer cell ablation

Photocatalytic cancer cell elimination, as an emerging therapeutic strategy, has demonstrated immense potential and broad applicability. However, numerous challenges remain to be addressed. Traditional photocatalytic reactions predominantly rely on ultraviolet–visible (UV–Vis) light as the excitation source. Yet, the penetration depth of these wavelengths in human tissue is limited, typically only a few millimeters, restricting the effectiveness of photocatalytic therapy in treating deep–seated tumors. While near–infrared (NIR) light offers greater tissue penetration, NIR–based photodynamic therapy depends on the generation of cytotoxic ROS, such as singlet oxygen, which requires oxygen. This dependency may be less effective in hypoxic tumor environments, further diminishing therapeutic efficacy. Additionally, upon light irradiation, the excited–state valence band holes and conduction band electrons in photocatalysts are prone to rapid recombination or surface trapping, resulting in low photocatalytic efficiency and suboptimal therapeutic outcomes. In response, researchers have introduced S–scheme heterojunctions, OVs, and multi–cooperative effects of noble metal ions to significantly enhance photocatalytic performance. However, these high–efficiency nanophotocatalysts still suffer from a lack of selectivity, potentially damaging healthy cells while targeting cancer cells. Therefore, improving the selectivity of photocatalysts toward cancer cells has become a crucial research focus.

Moreover, the stability and biocompatibility of photocatalysts within biological systems present another major challenge for nanophotocatalytic cancer cell elimination. Researchers must ensure that photocatalysts do not elicit immune or toxic reactions within the body. Although preliminary studies suggest that certain photocatalysts exhibit low toxicity, these investigations are often limited to short–term observations. Long–term toxicity assessments are critically important and require rigorous animal and human trials to validate their safety. Optimizing the photocatalytic treatment protocols also remains a pivotal task. Scientists must determine the optimal light intensity, wavelength, irradiation duration, and dosage to achieve the best therapeutic effects while minimizing adverse impacts on healthy tissues. Despite these challenges, nanophotocatalysts have shown the capability to generate large quantities of ROS (e.g., **·**OH, superoxide anions) under specific wavelengths of light. These ROS can penetrate cell membranes, inducing oxidative damage in tumor cells, leading to apoptosis or necrosis. Importantly, these nanophotocatalysts tend to exhibit relatively low toxicity toward normal cells, playing a significant role in the precision treatment of cancer.

**Figure 4:**
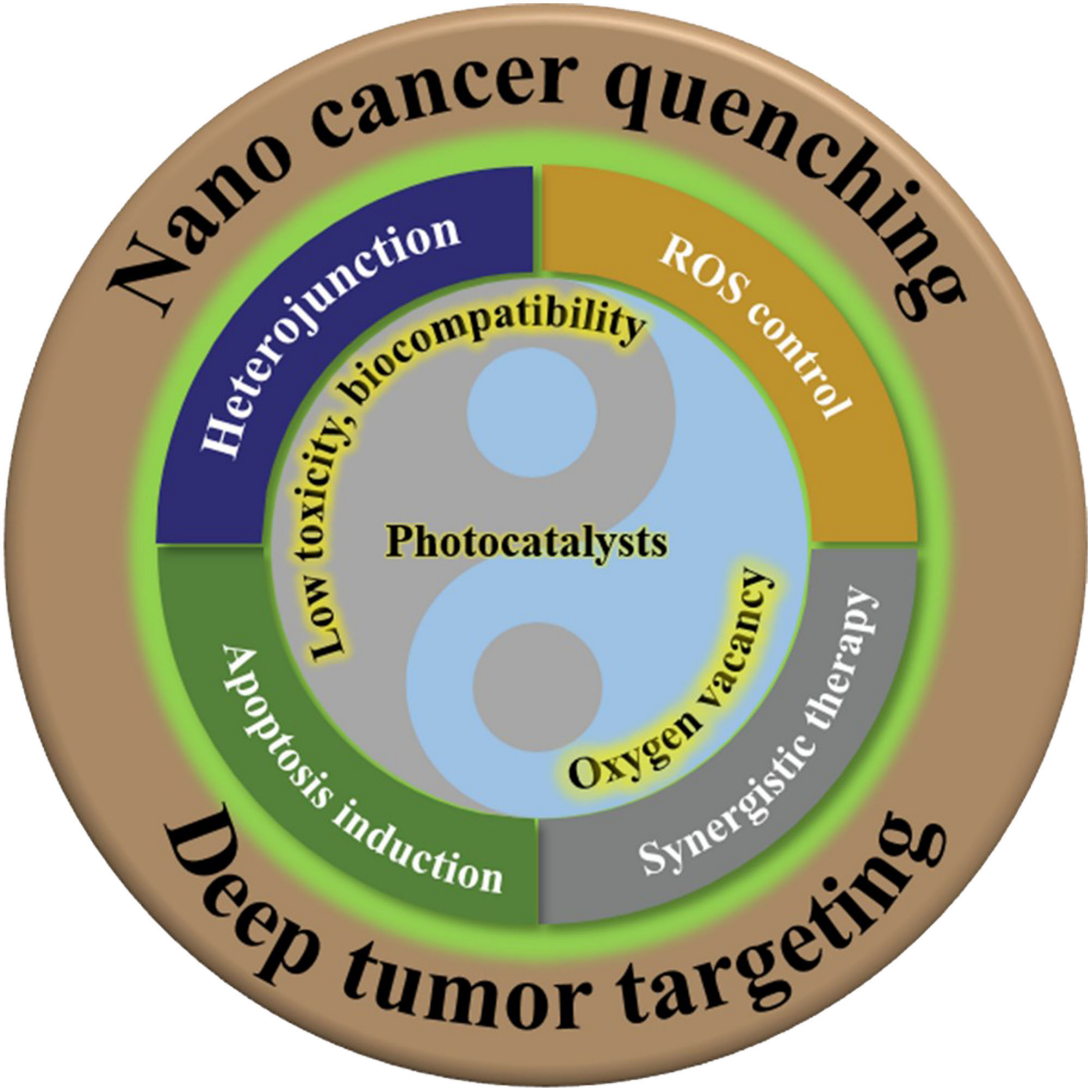
Tumor clearance strategy based on nano photocatalysis technology.

With the rapid advancements in materials science, nanotechnology, and biomedical engineering, the application of nanophotocatalysts with high photocatalytic activity and low toxicity in cancer treatment will become more widespread and profound (shown in **Figure 4**). Future research directions may include: (I) the development of intelligent responsive photocatalysts, which exhibit enhanced photocatalytic activity under specific conditions by incorporating temperature–, pH–, or light–sensitive groups, thereby increasing the precision of treatment and enabling on–demand release of therapeutic agents *in vivo* to minimize unwanted side effects; (II) the integration of multimodal therapeutic strategies, combining photocatalytic therapy with other treatments (e.g., chemotherapy, immunotherapy, sonodynamic therapy, photothermal therapy) to achieve a more comprehensive therapeutic outcome and reduce the risk of recurrence; and (III) the development of precise delivery systems, utilizing targeted molecular modifications, optimization of nanoparticle size and shape, and the assistance of external fields (e.g., magnetic or ultrasonic fields) to ensure accurate delivery of nanophotocatalysts to the tumor site and efficient release of therapeutic agents. These future directions will foster more innovative breakthroughs in cancer treatment, offering new perspectives and possibilities for the application of photocatalytic technology in medicine.

## 5 Conclusion

Nanophotocatalytic technology, as an emerging cancer treatment strategy, has achieved remarkable progress in scientific research in recent years. This technology utilizes nanophotocatalysts to generate reactive oxygen species (ROS) under light excitation, enabling precise targeting and effective elimination of cancer cells. This review primarily explores how the use of highly efficient nanophotocatalysts and various synergistic mechanisms, such as S–scheme heterojunctions and oxygen vacancies (OVs), can enhance light absorption efficiency and reduce the electron–hole recombination rates, thus improving photocatalytic performance. Through these mechanisms, the photocatalytic reaction can significantly increase ROS generation, resulting in the precise destruction and effective elimination of cancer cells. Furthermore, the nanophotocatalysts employed in photocatalytic technology not only demonstrate exceptional photocatalytic efficiency and selectivity but also minimize adverse effects on healthy tissues, enhancing overall therapeutic outcomes and offering new hope for cancer treatment. Simultaneously, researchers continue to explore and optimize the types, structures, and properties of nanomaterials to further enhance their photocatalytic efficiency and biocompatibility, accelerating the clinical application of nanophotocatalytic cancer treatment and providing safer and more effective therapeutic options for cancer patients.

## References

[1] Rizwan M Ali S Qayyum MF Sik Ok Y Adrees M Ibrahim M. Effect of metal and metal oxide nanoparticles on growth and physiology of globally important food crops: A critical review. J Hazard Mater. (2017) 322:2–16. doi: 10.1016/j.jhazmat.2016.05.061 27267650

[2] Rizwan M Ali S Qayyum MF Sik Ok Y Zia–ur–Rehman M Abbas Z. Use of maize (Zea mays L.) for phytomanagement of Cd–contaminated soils: a critical review. Environ Geochem Hlth. (2017) 39:259–77. doi: 10.1007/s10653-016-9826-0 27061410

[3] Tahir MB Kiran H Iqbal T. The detoxification of heavy metals from aqueous environment using nano–photocatalysis approach: a review. Environ Sci pollut R. (2019) 26:10515–28. doi: 10.1007/s11356-019-04547-x 30835072

[4] Wu XH Chen GQ Wang J Li JM Wang GH. Review on S–scheme heterodjunctions for photocatalytic hydrogen evolution. Acta Phys–Chim Sin. (2023) 39:2212016. doi: 10.3866/PKU.WHXB202212016

[5] Xiao YW Tian X Chen YH Xiao XC Chen T Wang YD. Recent advances in carbon nitride–based S–scheme photocatalysts for solar energy conversion. Materials. (2023) 16:3745. doi: 10.3390/ma16103745 37241371 PMC10223283

[6] Yuan L Du PY Yin LL Yao JM Wang J Liu C. Metal–organic framework–based S–scheme heterojunction photocatalysts. Nanoscale. (2024) 16:5487–503. doi: 10.1039/d3nr06677k 38393670

[7] Li Z Li W Zhai LG Chen CX Zhang JF Wang ZH. Oxygen defects and S–scheme heterojunctions synergistically promote the photocatalytic hydrogen evolution activity and stability of WO_2.72_/Zn_0.5_Cd_0.5_S–DETA nanocomposites. J Colloid Interf Sci. (2023) 646:834–43. doi: 10.1016/j.jcis.2023.05.084 37230001

[8] Zhang LY Zhang JJ Yu HG Yu JG. Emerging S–scheme photocatalyst. Adv Mater. (2022) 34:2107668. doi: 10.1002/adma.202107668 34962659

[9] Li Z Liu W Chen CX Ma TT Zhang JF Wang ZH. Transforming the charge transfer mechanism in the In_2_O_3_/CdSe–DETA nanocomposite from Type–I to S–scheme to improve photocatalytic activity and stability during hydrogen production. Acta Phys–Chim Sin. (2023) 39:2208030. doi: 10.3866/PKU.WHXB202208030

[10] Li YF Xia ZL Yang Q Wang LX Xing Y. Review on g–C_3_N_4_–based S–scheme heterojunction photocatalysts. J Mater Sci Technol. (2022) 125:128–44. doi: 10.1016/j.jmst.2022.02.035

[11] Wang ZM Yue XY Xiang QJ. MOFs–based S–scheme heterojunction photocatalysts. Coordin Chem Rev. (2024) 504:215674. doi: 10.1016/j.ccr.2024.215674

[12] Li FY Zhu GH Jiang JZ Yang L Deng FX Arramel. A review of updated S–scheme heterojunction photocatalysts. J Mater Sci Technol. (2023) 177:142–80. doi: 10.1016/j.jmst.2023.08.038

[13] Zhang FB Wang XM Liu HN Liu CL Wan Y Long YZ. Recent advances and applications of semiconductor photocatalytic technology. Appl Sci. (2019) 9:2489. doi: 10.3390/app9122489

[14] Geng Q Wang H Chen RM Chen LC Li KL Dong F. Advances and challenges of photocatalytic technology for air purification. Natl Sci Open. (2022) 1:20220025. doi: 10.1360/nso/20220025

[15] Li T Tsubaki N Jin ZL. S–scheme heterojunction in photocatalytic hydrogen production. J Mater Sci Technol. (2024) 169:82–104. doi: 10.1016/j.jmst.2023.04.049

[16] Lu JN Gu SN Li HD Wang YN Guo M Zhou GW. Review on multi–dimensional assembled S–scheme heterojunction photocatalysts. J Mater Sci Technol. (2023) 160:214–39. doi: 10.1016/j.jmst.2023.03.027

[17] Nazir A Huo PW Rasool AT. Recent advances on graphitic carbon nitride–based S–scheme photocatalysts: synthesis, environmental applications, and challenges. J Organomet Chem. (2024) 1004:122951. doi: 10.1016/j.jorganchem.2023.122951

[18] Nie CL Wang XH Lu P Zhu YK Li X Tang H. Advancements in S–scheme heterojunction materials for photocatalytic environmental remediation. J Mater Sci Technol. (2024) 169:182–98. doi: 10.1016/j.jmst.2023.06.011

[19] Ren YJ Li YF Pan GX Wang N Xing Y Zhang ZY. Recent progress in CdS–based S–scheme photocatalysts. J Mater Sci Technol. (2024) 171:162–84. doi: 10.1016/j.jmst.2023.06.052

[20] Bayda S Adeel M Tuccinardi T Cordani M Rizzolio F. The history of nanoscience and nanotechnology: from chemical–physical applications to nanomedicine. Molecules. (2019) 25:112. doi: 10.3390/molecules25010112 31892180 PMC6982820

[21] Yang BW Chen Y Shi JL. Nanocatalytic medicine. Adv Mater. (2019) 31:1901778. doi: 10.1002/adma.201901778 31328844

[22] Prakash J Cho J Mishra YK. Photocatalytic TiO_2_ nanomaterials as potential antimicrobial and antiviral agents: Scope against blocking the SARS–COV–2 spread. Micro Nano Eng. (2022) 14:100100. doi: 10.1016/j.mne.2021.100100 PMC8685168 40477493

[23] Prakash J Sun SH Swart HC Gupta RK. Noble metals–TiO_2_ nanocomposites: from fundamental mechanisms to photocatalysis, surface enhanced Raman scattering and antibacterial applications. Appl Mater Today. (2018) 11:82–135. doi: 10.1016/j.apmt.2018.02.002

[24] Zeghioud H Assadi AA Khellaf N Djelal H Amrane A Rtimi S. Photocatalytic performance of Cu_x_O/TiO_2_ deposited by HiPIMS on polyester under visible light LEDs: Oxidants, ions effect, and reactive oxygen species investigation. Materials. (2019) 12:412. doi: 10.3390/ma12030412 30699939 PMC6385099

[25] Bai HW Liu ZY Liu L Sun DD. Large–scale production of hierarchical TiO_2_ nanorod spheres for photocatalytic elimination of contaminants and killing bacteria. Chem–Eur J. (2013) 19:3061–70. doi: 10.1002/chem.201204013 23362129

[26] Manoharan D Wang LC Chen YC Li WP Yeh CS. Catalytic nanoparticles in biomedical applications: exploiting advanced nanozymes for therapeutics and Diagnostics. Adv Healthc Mater. (2024) 13:2400746. doi: 10.1002/adhm.202400746 38683107

[27] Sharma A Goyal AK Rath G. Recent advances in metal nanoparticles in cancer therapy. J Drug Targeting. (2018) 26:617–32. doi: 10.1080/1061186X.2017.1400553 29095640

[28] Padma VV. An overview of targeted cancer therapy. BioMedicine. (2015) 5:1–6. doi: 10.7603/s40681-015-0019-4 26613930 PMC4662664

[29] Mathur G Nain S Sharma PK. Cancer: an overview. Acad J Cancer Res. (2015) 8:01–9. doi: 10.5829/idosi.ajcr.2015.8.1.9336

[30] Zugazagoitia J Guedes C Ponce S Ponce C Ferrer I Molina–Pinelo S. Current challenges in cancer treatment. Clin Ther. (2016) 38:1551–66. doi: 10.1016/j.clinthera.2016.03.026 27158009

[31] Xiang HJ Chen Y. Energy–converting nanomedicine. Small. (2019) 15:1805339. doi: 10.1002/smll.201805339 30773837

[32] Li BL Zhao SJ Huang L Wang Q Xiao JF Lan MH. Recent advances and prospects of carbon dots in phototherapy. Chem Eng J. (2021) 408:127245. doi: 10.1016/j.cej.2020.127245

[33] Noh I Lee DY Kim H Jeong CU Lee Y Ahn JO. Enhanced photodynamic cancer treatment by mitochondria–targeting and brominated near–infrared fluorophores. Adv Sci. (2018) 5:1700481. doi: 10.1002/advs.201700481 PMC5867131 29593951

[34] Jukapli NM Bagheri S. Recent developments on titania nanoparticle as photocatalytic cancer cells treatment. J Photoch Photobio B. (2016) 163:421–30. doi: 10.1016/j.jphotobiol.2016.08.046 27639172

[35] Li WB Wang C Yao YF Wu CP Luo WJ Zou ZG. Photocatalytic materials: an apollo’s arrow to tumor cells. Trends Chem. (2020) 2:1126–40. doi: 10.1016/j.trechm.2020.10.002

[36] Zou MZ Liu WL Chen HS Bai XF Gao F Ye JJ. Advances in nanomaterials for treatment of hypoxic tumor. Natl Sci Rev. (2021) 8:nwaa160. doi: 10.1093/nsr/nwaa160 34691571 PMC8288333

[37] Hu X Wang N Guo X Liang ZY Sun H Liao HW. A sub–nanostructural transformable nanozyme for tumor photocatalytic therapy. Nano–Micro Lett. (2022) 14:101. doi: 10.1007/s40820-022-00848-y PMC9005554 35412159

[38] Bonet–Aleta J Garcia–Peiro JI Hueso JL. Engineered nanostructured photocatalysts for cancer therapy. Catalysts. (2022) 12:167. doi: 10.3390/catal12020167

[39] Neubauer A Grell G Friedrich A Bokarev SI Schwarzbach P Gartner F. Electron–and energy–transfer processes in a photocatalytic system based on an Ir (III)–photosensitizer and an iron catalyst. J Phys Chem Lett. (2014) 5:1355–60. doi: 10.1021/jz5004318 26269979

[40] Radhika NP Selvin R Kakkar R Umar A. Recent advances in nano–photocatalysts for organic synthesis. Arab J Chem. (2019) 12:4550–78. doi: 10.1016/j.arabjc.2016.07.007

[41] Fresno F Portela R Suárez S Coronado JM. Photocatalytic materials: recent achievements and near future trends. J Mater Chem A. (2014) 2:2863–84. doi: 10.1039/b000000x

[42] Joseph A Vijayanandan A. Review on support materials used for immobilization of nano–photocatalysts for water treatment applications. Inorg Chim Acta. (2023) 545:121284. doi: 10.1016/j.ica.2022.121284

[43] Mehta M Chopra L Manikanika. Applications of nano photocatalysts in the degradation of biomedical waste: A short review. Mater Today Proc. (2022) 68:695–700. doi: 10.1016/j.matpr.2022.05.563

[44] Prakash J Krishna SBN Kumar P Kumar V Ghosh KS Swart HC. Recent advances on metal oxide based nano–photocatalysts as potential antibacterial and antiviral agents. Catalysts. (2022) 12:1047. doi: 10.3390/catal12091047

[45] Khatami M Iravani S. Green and eco–friendly synthesis of nanophotocatalysts: an overview. Comment Inorg Chem. (2021) 41:133–87. doi: 10.1080/02603594.2021.1895127

[46] Li W Chen CX Yang RQ Cheng SL Sang XY Zhang MW. Efficient and stable degradation of triazophos pesticide by TiO_2_/WO_3_ nanocomposites with S–scheme heterojunctions and oxygen defects. Catalysts. (2023) 13:1136. doi: 10.3390/catal13071136

[47] Ma J Long R Liu D Low JX Xiong YJ. Defect engineering in photocatalytic methane conversion. Small Struct. (2022) 3:2100147. doi: 10.1002/sstr.202100147

[48] Zhao YF Mao QY Zhai XY Zhang GY. Structural defects regulation of bismuth molybdate photocatalyst. Prog Chem. (2021) 33:1331–43. doi: 10.7536/PC201236

[49] Sultana S Mansingh S Parida KM. Crystal facet and surface defect engineered low dimensional CeO_2_ (0D, 1D, 2D) based photocatalytic materials towards energy generation and pollution abatement. Mater Adv. (2021) 2:6942–83. doi: 10.1039/d1ma00539a

[50] Zhang JJ Zhang LY Wang W Yu JG. *In situ* irradiated X–ray photoelectron spectroscopy investigation on electron transfer mechanism in S–scheme photocatalyst. J Phys Chem Lett. (2022) 13:8462–9. doi: 10.1021/acs.jpclett.2c02125 36053788

[51] Yang H Zhao ZC Yang YP Zhang Z Chen W Yan RQ. Defective WO_3_ nanoplates controllably decorated with MIL–101 (Fe) nanoparticles to efficiently remove tetracycline hydrochloride by S–scheme mechanism. Sep Purif Technol. (2022) 300:121846. doi: 10.1016/j.seppur.2022.121846

[52] Cheng C Zhang JJ Zhu BC Liang GJ Zhang LY Yu JG. Verifying the charge–transfer mechanism in S–scheme heterojunctions using femtosecond transient absorption spectroscopy. Angew Chem Int Edit. (2023) 62:e202218688. doi: 10.1002/anie.202218688 36579457

[53] Tuli HS Kaur J Vashishth K Sak K Sharma U Choudhary R. Molecular mechanisms behind ROS regulation in cancer: a balancing act between augmented tumorigenesis and cell apoptosis. Arch Toxicol. (2023) 97:103–20. doi: 10.1007/s00204-022-03421-z 36443493

[54] Nakamura H Takada K. Reactive oxygen species in cancer: current findings and future directions. Cancer Sci. (2021) 112:3945–52. doi: 10.1111/cas.15068 PMC8486193 34286881

[55] Kwon S Ko H You DG Kataoka K Park J. Nanomedicines for reactive oxygen species mediated approach: an emerging paradigm for cancer treatment. Acc Chem Res. (2019) 52:1771–82. doi: 10.1021/acs.accounts.9b00136 31241894

[56] Aggarwal V Tuli HS Varol A Thakral F Yerer MB Sak K. Role of reactive oxygen species in cancer progression: molecular mechanisms and recent advancements. Biomolecules. (2019) 9:735. doi: 10.3390/biom9110735 31766246 PMC6920770

[57] Wu K Zowalaty AEE Sayin VI Papagiannakopoulos T. The pleiotropic functions of reactive oxygen species in cancer. Nat Cancer. (2024) 5:384–99. doi: 10.1038/s43018-024-00738-9 38531982

[58] Malhotra K Malik A Almalki WH Sahebkar A Kesharwani P. Reactive oxygen species and its manipulation strategies in cancer treatment. Curr Med Chem. (2024) 32:55–73. doi: 10.2174/0929867330666230609110455 37303173

[59] Njema G Kibet JK. A review of novel materials for nano–photocatalytic and optoelectronic applications: recent perspectives, water splitting and environmental remediation. Prog Eng Sci. (2024), 1: 100018. doi: 10.1016/j.pes.2024.100018

[60] Zekic E Vukovic Z Halkijevic I. Application of nanotechnology in wastewater treatment. Građevinar. (2017) 69:225–30. doi: 10.14256/JCE.2165.2017

[61] Li W Meng AY Li CS Sun Y Zhang JF Li Z. Enhanced efficiency and stability in the degradation of triazophosphorus pesticides by Al_6_Si_2_O_13_/WO_2.72_ nanocomposites through synergistic action of S–scheme heterojunction and oxygen vacancies. J Colloid Interf Sci. (2025) 677:704–17. doi: 10.1016/j.jcis.2024.07.240 39116568

[62] Li W Wu ZH Meng AY Li ZH Chen XD Zhang JF. Efficient and stable photocatalytic degradation of dichlorvos using an S–scheme ZnO/WO_2.72_ nanocomposite. Mater Lett. (2025) 382:137867. doi: 10.1016/j.matlet.2024.137867

[63] Aebisher D Woźnicki P Bartusik–Aebisher D. Photodynamic therapy and adaptive immunity induced by reactive oxygen species: recent reports. Cancers. (2024) 16:967. doi: 10.3390/cancers16050967 38473328 PMC10930503

[64] He M Wang MY Xu T Zhang MY Dai HX Wang C. Reactive oxygen species–powered cancer immunotherapy: current status and challenges. J Control Release. (2023) 356:623–48. doi: 10.1016/j.jconrel.2023.02.040 36868519

[65] Zhao YX Ye XC Xiong ZF Ihsan A Ares I Martínez M. Cancer metabolism: the role of ROS in DNA damage and induction of apoptosis in cancer cells. Metabolites. (2023) 13:796. doi: 10.3390/metabo13070796 37512503 PMC10383295

[66] Liberto GD Cipriano LA Tosoni S Pacchioni G. Rational design of semiconductor heterojunctions for photocatalysis. Chem Eur J. (2021) 27:13306–17. doi: 10.1002/chem.202101764 PMC8518984 34264526

[67] Sun CJ Zhao LP Wang R. Recent advances in heterostructured photocatalysts for degradation of organic pollutants. Mini–Rev Org Chem. (2021) 18:649–69. doi: 10.2174/1570193X17999200820161301

[68] Zhou X Wu J Li QF Zeng T Ji Z He P. Carbon decorated In_2_O_3_/TiO_2_ heterostructures with enhanced visible–light–driven photocatalytic activity. J Catal. (2017) 355:26–39. doi: 10.1016/j.jcat.2017.09.006

[69] Sa–nguanprang S Phuruangrat A Thongtem T Thongtem S. Characterization and photocatalysis of visible–light–driven Dy–doped ZnO nanoparticles synthesized by tartaric acid–assisted combustion method. Inorg Chemi Commun. (2020) 117:107944. doi: 10.1016/j.inoche.2020.107944

[70] Jie LF Gao X Cao XQ Wu S Long XX Ma QY. A review of CdS photocatalytic nanomaterials: Morphology, synthesis methods, and applications. Mat Sci Semicon Proc. (2024) 176:108288. doi: 10.1016/j.mssp.2024.108288

[71] Mishra SR Gadore V Ahmaruzzaman M. An overview of In_2_S_3_ and In_2_S_3_–based photocatalyst: characteristics, synthesis, modifications, design strategies, and catalytic environmental application. J Environ Chem Eng. (2024) 12:113449. doi: 10.1016/j.jece.2024.113449

[72] Zhao B Wang YS Yao XX Chen DY Fan MJ Jin ZK. Photocatalysis–mediated drug–free sustainable cancer therapy using nanocatalyst. Nat Commun. (2021) 12:1345. doi: 10.1038/s41467-021-21618-1 33649319 PMC7921091

[73] Ge CY Yang EQ Zhao XZ Yuan C Li S Dong C. Efficient Near–infrared PbS quantum dot solar cells employing hydrogenated In_2_O_3_ transparent electrode. Small. (2022) 18:2203677. doi: 10.1002/smll.202203677 36148851

[74] Shah S Hussain S Din STU Shahid A Amu–Darko JNO Wang MS. A review on In_2_O_3_ nanostructures for gas sensing applications. J Environ Chem Eng. (2024) 12:112538. doi: 10.1016/j.jece.2024.112538

[75] Park HG Hussain SQ Park J Yi J. Influence of hydrogen doping of In_2_O_3_–based transparent conducting oxide films on silicon heterojunction solar cells. J Mater Sci. (2024) 59:13873–82. doi: 10.1007/s10853-024-09506-7

[76] Alaizeri ZM Alhadlaq HA Akhtar MJ Aldawood S. Zn–modified In_2_O_3_ nanoparticles: facile synthesis, characterization, and selective cytotoxicity against human cancer cells. J King Saud Univ–Sci. (2024) 36:103015. doi: 10.1016/j.jksus.2023.103015

[77] Duan YL Xue JQ Dai JN Wei YR Wu C Chang SH. Interface engineering of ZnO/In_2_O_3_ Z–scheme heterojunction with yolk–shell structure for efficient photocatalytic hydrogen evolution. Appl Surf Sci. (2022) 592:153306. doi: 10.1016/j.apsusc.2022.153306

[78] Wang Q Chen YJ Liu X Li LG Du LZ Tian GH. Sulfur doped In_2_O_3_–CeO_2_ hollow hexagonal prisms with carbon coating for efficient photocatalytic CO_2_ reduction. Chem Eng J. (2021) 421:129968. doi: 10.1016/j.cej.2021.129968

[79] Habib A Khan MS Zubair M Ul Hasan I. Ni–Doped In_2_O_3_ nanoparticles and their composite with rGO for efficient degradation of Organic pollutants in Wastewater under visible light irradiation. Int J Mol Sci. (2023) 24:7950. doi: 10.3390/ijms24097950 37175664 PMC10178878

[80] Chang P Wang YH Wang YT Zhu YY. Current trends on In_2_O_3_ based heterojunction photocatalytic systems in photocatalytic application. Chem Eng J. (2022) 450:137804. doi: 10.1016/j.cej.2022.137804

[81] Han L Jing F Zhang J Luo XZ Zhong YL Wang K. Environment friendly and remarkably efficient photocatalytic hydrogen evolution based on metal organic framework derived hexagonal/cubic In_2_O_3_ phase–junction. Appl Catal B–Environ. (2021) 282:119602. doi: 10.1016/j.apcatb.2020.119602

[82] Li W Yang RQ Zhai LG Meng QB Wang ZH Zhang JF. Highly efficient photocatalytic decomposition of triazophos using novel In_2_O_3_/WO_3_ nanocomposites with oxygen defects and S–scheme heterojunctions. Int J Hydrogen Energ. (2024) 57:369–78. doi: 10.1016/j.ijhydene.2024.01.061

[83] Du H Akakuru OU Yao CY Yang F Wu AG. Transition metal ion–doped ferrites nanoparticles for bioimaging and cancer therapy. Transl Oncol. (2022) 15:101264. doi: 10.1016/j.tranon.2021.101264 34781185 PMC8593663

[84] Fu CH Zhou HQ Tan LF Huang ZB Wu Q Ren XL. Microwave–activated Mn–doped zirconium metal–organic framework nanocubes for highly effective combination of microwave dynamic and thermal therapies against cancer. ACS Nano. (2017) 12:2201–10. doi: 10.1021/acsnano.7b08868 29286623

[85] Zhang JW Shi CX Mahmood N Ai M Pan L Huang ZF. Recent progress on the development of carbon nitride based all–solid Z–scheme photocatalyst for solar energy conversion applications. Energy Technol–Ger. (2022) 10:2000950. doi: 10.1002/ente.202000950

[86] Khosroshahi N Goudarzi MD Safarifard V. Fabrication of a novel heteroepitaxial structure from an MOF–on–MOF architecture as a photocatalyst for highly efficient Cr (vi) reduction. New J Chem. (2022) 46:3106–15. doi: 10.1039/d1nj05440f

[87] Dong WB Xiao YY Qin ZY Qiao B Li LY. Partially H–bonded covalent organic frameworks for photocatalytic hydrogen evolution. J Mater Chem A. (2023) 11:14760–7. doi: 10.1039/d3ta01944f

[88] Cai MJ Wang CC Liu YP Yan RY Li SJ. Boosted photocatalytic antibiotic degradation performance of Cd_0.5_Zn_0.5_S/carbon dots/Bi_2_WO_6_ S–scheme heterojunction with carbon dots as the electron bridge. Sep Purif Technol. (2022) 300:121892. doi: 10.1016/j.seppur.2022.121892

[89] Kočí K Reli M Troppová I Sihor M Kupková J Kustrowski P. Photocatalytic decomposition of N_2_O over TiO_2_/g–C_3_N_4_ photocatalysts heterojunction. Appl Surf Sci. (2017) 396:1685–95. doi: 10.1016/j.apsusc.2016.11.242

[90] Zhu YJ Wang L Xu WT Xu ZH Yuan JS Zhang GL. ZnO/Cu_2_O/g–C_3_N_4_ heterojunctions with enhanced photocatalytic activity for removal of hazardous antibiotics. Heliyon. (2022) 8:e12644. doi: 10.1016/j.heliyon.2022.e12644 36643305 PMC9834774

[91] Qi SY Guan L Zhang RY Wu SQ Zhang KY. Study on the photocatalytic degradation of rhodamine B by g–C_3_N_4_/Bi_2_Fe_4_O_9_ heterojunction photocatalyst. J Inorg Organomet Chem. (2023) 33:3675–83. doi: 10.1007/s10904-023-02800-y

[92] Chen SF Hu YF Jiang XL Meng SG Fu XL. Fabrication and characterization of novel Z–scheme photocatalyst WO_3_/g–C_3_N_4_ with high efficient visible light photocatalytic activity. Mater Chem Phys. (2015) 149:512–21. doi: 10.1016/j.matchemphys.2014.11.001

[93] Tan CE Lee JT Su EC Wey MY. Facile approach for Z–scheme type Pt/g–C_3_N_4_/SrTiO_3_ heterojunction semiconductor synthesis via low–temperature process for simultaneous dyes degradation and hydrogen production. Int J Hydrogen Energ. (2020) 45:13330–9. doi: 10.1016/j.ijhydene.2020.03.034

[94] Ni ZT Ding SY Zhang H Dai RJ Chen AR Wang RF. Phosphorus and selenium co–doped WO_3_ nanoparticles for interface modification and photovoltaic properties enhancement of monolayer planar Silicon/PEDOT: PSS hybrid solar cells. Adv Mater Interfaces. (2022) 9:2200812. doi: 10.1002/admi.202200812

[95] Enesca A Isac L. Tuned S–scheme Cu_2_S/TiO_2_/WO_3_ heterostructure photocatalyst toward S–metolachlor (S–MCh) herbicide removal. Materials. (2021) 14:2231. doi: 10.3390/ma14092231 33926016 PMC8123602

[96] Zhao D Chen CC Yu CL Ma WH Zhao JC. Photoinduced electron storage in WO_3_/TiO_2_ nanohybrid material in the presence of oxygen and postirradiated reduction of heavy metal ions. J Phys Chem C. (2009) 113:13160–5. doi: 10.1021/jp9002774

[97] Prabhu S Cindrella L Kwon OJ Mohanraju K. Photoelectrochemical and photocatalytic activity of TiO_2_/WO_3_ heterostructures boosted by mutual interaction. Mater Sci Semicond Process. (2018) 88:10–9. doi: 10.1016/j.mssp.2018.07.028

[98] Tatsuma T Takeda S Saitoh S Ohko Y Fujishima A. Bactericidal effect of an energy storage TiO_2_/WO_3_ photocatalyst in dark. Electrochem Commun. (2003) 5:793–6. doi: 10.1016/j.elecom.2003.07.003

[99] Ahmad A Sharif H Luque R Alsaiari M Harraz FA. 10 Light–driven photocatalysis using biomaterials for biomedical applications. Biomater Photocatal Gruyter. (2023) 6:8. doi: 10.1515/9783110768749-010

[100] Ahmad I Shukrullah S Naz MY Ahmed E Ahmad M Obaidullah AJ. An aimed review of current advances, challenges, and future perspectives of TiO_2_–based S–scheme heterojunction photocatalysts. Mater Sci Semicond Process. (2024) 172:108088. doi: 10.1016/j.mssp.2023.108088

[101] Habibi M Habibi–Yangjeh A Khataee A. S–scheme CeO_2–x_/AgFeO_2_/Ag photocatalysts with impressive activity in degradation of different antibiotics under visible light. Surf Interfaces. (2023) 39:102937. doi: 10.1016/j.surfin.2023.102937

[102] Yang RJ Mei L Fan YY Zhang QY Zhu RS Amal R. ZnIn_2_S_4_–based photocatalysts for energy and environmental applications. Small Methods. (2021) 5:2100887. doi: 10.1002/smtd.202100887 34927932

[103] Wu XH Chen GQ Wang J Li JM Wang GH. Review on S–scheme heterojunctions for photocatalytic hydrogen evolution. Acta Phys.–Chim Sin. (2023) 39:221201. doi: 10.3866/PKU.WHXB202212016

[104] Hakimi–Tehrani MJ Hassanzadeh–Tabrizi SA Koupaei N Saffar A Rafiei M. Synthesis of Z–scheme g–C_3_N_4_/WO_3_ nano–photocatalyst with superior antibacterial characteristics for wastewater treatmen. J Sol–Gel Sci Techn. (2023) 105:212–9. doi: 10.1007/s10971-022-05985-9

[105] Duan GX Chen L Jing ZF Luna PD Wen L Zhang LL. Robust antibacterial activity of tungsten oxide (WO_3–x_) nanodots. Chem Res Toxicol. (2019) 32:1357–66. doi: 10.1021/acs.chemrestox.8b00399 31251039

[106] Leifels M Cheng D Sozzi E Shoults DC Wuertz S Mongkolsuk S. Capsid integrity quantitative PCR to determine virus infectivity in environmental and food applications–a systematic review. Water Res X. (2021) 11:100080. doi: 10.1016/j.wroa.2020.100080 33490943 PMC7811166

[107] Zhang C Xiong W Li Y Lin L Zhou XY Xiong XY. Continuous inactivation of human adenoviruses in water by a novel g–C_3_N_4_/WO_3_/biochar memory photocatalyst under light–dark cycles. J Hazard Mater. (2023) 442:130013. doi: 10.1016/j.jhazmat.2022.130013 36155297

[108] Ferrari M. Cancer nanotechnology: opportunities and challenges. Nat Rev Cancer. (2005) 5:161–71. doi: 10.1038/nrc1566 15738981

[109] Sengupta S Sasisekharan R. Exploiting nanotechnology to target cancer. Brit J Cancer. (2007) 96:1315–9. doi: 10.1038/sj.bjc.6603707 PMC2360169 17406364

[110] Zhang ZJ Wang L Liu WX Yan ZH Zhu YF Zhou SY. Photogenerated–hole–induced rapid elimination of solid tumors by the supramolecular porphyrin photocatalyst. Natl Sci Rev. (2021) 8:nwaa155. doi: 10.1093/nsr/nwaa155 34691632 PMC8288340

[111] Wang SY Chen WH Jiang CH Lu LH. Nano scaled porphyrinic metal–organic framework for photodynamic/photothermal therapy of tumor. Electrophoresis. (2019) 40:2204–10. doi: 10.1002/elps.201900005 30953373

[112] Li B Wang XY Chen L Zhou YL Dang WT Chang J. Ultrathin Cu–TCPP MOF nanosheets: a new theragnostic nanoplatform with magnetic resonance/near–infrared thermal imaging for synergistic phototherapy of cancers. Theranostics. (2018) 8:4086–96. doi: 10.7150/thno.25433 PMC6096389 30128038

[113] Chen WD Zhao JK Hou MF Yang M Yi CQ. Gadolinium–porphyrin based polymer nanotheranostics for fluorescence/magnetic resonance imaging guided photodynamic therapy. Nanoscale. (2021) 13:16197–206. doi: 10.1039/D1NR04489C 34545903

[114] Shi ZJ Zhang K Zada S Zhang C Meng XD Yang Z. Upconversion nanoparticle–induced multimode photodynamic therapy based on a metal–organic framework/titanium dioxide nanocomposite. ACS Appl Mate Interfaces. (2020) 12:12600–8. doi: 10.1021/acsami.0c01467 32096623

[115] Yin SY Song GS Yang Y Zhao Y Wang P Zhu LM. Persistent regulation of tumor microenvironment via circulating catalysis of MnFe_2_O_4_@ metal–organic frameworks for enhanced photodynamic therapy. Adv Funct Mater. (2019) 29:1901417. doi: 10.1002/adfm.201901417

[116] Li W Meng AY Tian XH Ye MF Zhang JF Li Z. Efficient and stable photocatalytic degradation of triazophos pesticides by g–C_3_N_4_/WO_2.72_ nano–composite with S–scheme heterojunction and oxygen vacancies. J Environ Chem Eng. (2024) 12:113587. doi: 10.1016/j.jece.2024.113587

[117] Chen RX Gan W Guo J Lu YQ Ding S Liu R. Internal electric field and oxygen vacancies synergistically boost S–scheme VO/BiOCl–TiO_2_ heterojunction film for photocatalytic degradation of norfloxacin. Chem Eng J. (2024) 489:151260. doi: 10.1016/j.cej.2024.151260

[118] Khan K Tao XP Shi M Zeng B Feng ZC Li C. Visible–light–driven photocatalytic hydrogen production on Cd_0.5_Zn_0.5_S nanorods with an apparent quantum efficiency exceeding 80%. Adv Funct Mater. (2020) 30:2003731. doi: 10.1002/adfm.202003731

[119] Yang JH Yan HJ Wang XL Wen FY Wang ZJ Fan DY. Roles of cocatalysts in Pt–PdS/CdS with exceptionally high quantum efficiency for photocatalytic hydrogen production. J Catal. (2012) 290:151–7. doi: 10.1016/j.jcat.2012.03.008

[120] Liu T Xiong Y Wang XY Xue YJ Liu WD Tian J. Dual cocatalysts and vacancy strategies for enhancing photocatalytic hydrogen production activity of Zn_3_In_2_S_6_ nanosheets with an apparent quantum efficiency of 66.20%. J Colloid Interf Sci. (2023) 640:31–40. doi: 10.1016/j.jcis.2023.02.043 36827846

[121] Shvalagin V Tarakina N Badamdorj B Lahrsenet IM Bargiacchi E Bardow A. Simultaneous photocatalytic production of H_2_ and acetal from ethanol with quantum efficiency over 73% by protonated poly (heptazine imide) under visible light. ACS Catal. (2024) 14:14836–54. doi: 10.1021/acscatal.4c04180 PMC11459976 39386918

[122] Jin YX Zheng DD Fang ZP Pan ZM Wang SB Hou YD. Salt–melt synthesis of poly (heptazine imide) in binary alkali metal bromides for enhanced visible–light photocatalytic hydrogen production. Interdiscip Mater. (2024) 3:389–99. doi: 10.1002/idm2.12159

[123] Rasmussen JW Martinez E Louka P Wingett DG. Zinc oxide nanoparticles for selective destruction of tumor cells and potential for drug delivery applications. Expert Opin Drug del. (2010) 7:1063–77. doi: 10.1517/17425247.2010.502560 PMC2924765 20716019

[124] Anjum S Hashim M Malik SA Khan M Lorenzo JM Abbasi BH. Recent advances in zinc oxide nanoparticles (ZnO NPs) for cancer diagnosis, target drug delivery, and treatment. Cancers. (2021) 13:4570. doi: 10.3390/cancers13184570 34572797 PMC8468934

[125] Shahbazi MA Faghfouri L Ferreira MPA Figueiredo P Maleki H Sefat F. The versatile biomedical applications of bismuth–based nanoparticles and composites: therapeutic, diagnostic, biosensing, and regenerative properties. Chem Soc Rev. (2020) 49:1253–321. doi: 10.1039/c9cs00283a 31998912

[126] Gao X Feng J Lv K Zhou YF Zhang RH Song SY. Engineering CeO_2_/CuO heterostructure anchored on upconversion nanoparticles with boosting ROS generation–primed apoptosis–ferroptosis for cancer dynamic therapy. Nano Res. (2023) 16:5322–34. doi: 10.1007/s12274-022-5223-4

[127] Lagos KJ Buzzá HH Bagnato VS Romero MP. Carbon–based materials in photodynamic and photothermal therapies applied to tumor destruction. Int J Mol Sci. (2021) 23:22. doi: 10.3390/ijms23010022 35008458 PMC8744821

[128] Zhang XW Luo LY Li L He YC Cao W Liu H. Trimodal synergistic antitumor drug delivery system based on graphene oxide. Nanomed–Nanotechnol. (2019) 15:142–52. doi: 10.1016/j.nano.2018.09.008 30300749

[129] Li QN Wang XM Lu XH Tian HE Jiang H Lv G. The incorporation of daunorubicin in cancer cells through the use of titanium dioxide whiskers. Biomaterials. (2009) 30:4708–15. doi: 10.1016/j.biomaterials.2009.05 19500830

[130] Pourmadadi M Rajabzadeh–Khosroshahi M Eshaghi MM Rahmani E Motasadizadeh H Arshad R. TiO_2_–based nanocomposites for cancer diagnosis and therapy: a comprehensive review. J Drug Delivery Sci Tec. (2023) 82:104370. doi: 10.1016/j.jddst.2023.104370

[131] Qin Y Sun L Li XX Cao QQ Wang H Tang XF. Highly water–dispersible TiO_2_ nanoparticles for doxorubicin delivery: effect of loading mode on therapeutic efficacy. J Mater Chem. (2011) 21:18003–10. doi: 10.1039/c1jm13615a

[132] Li KL Ma XT He S Wang L Yang XT Zhang GJ. Ultrathin nanosheet–supported Ag@Ag_2_O core–shell nanoparticles with vastly enhanced photothermal conversion efficiency for NIR–II–triggered photothermal therapy. ACS Biomater Sci Eng. (2022) 8:540–50. doi: 10.1021/acsbiomaterials.1c01291 35107009

[133] Shang LH Jiang X Yang T Xu HB Xie Q Hu M. Enhancing cancer chemo–immunotherapy by biomimetic nanogel with tumor targeting capacity and rapid drug–releasing in tumor microenvironment. Acta Pharm Sin B. (2022) 12:2550–67. doi: 10.1016/j.apsb.2021.11.004 PMC9136611 35646526

[134] Peng JL Du K Sun J Yang XL Wang X Zhang XR. Photocatalytic generation of hydrogen radical (H^·^) with GSH for photodynamic therapy. Angew Chem Int Edit. (2023) 62:e202214991. doi: 10.1002/anie.202214991 36537886

[135] Lan MH Zhao SJ Liu WM Lee CS Zhang WJ Wang PF. Photosensitizers for photodynamic therapy. Adv Healthc Mater. (2019) 8:1900132. doi: 10.1002/adhm.201900132 31067008

[136] Poß M Zittel E Seidl C Meschkov A Muñoz L Schepers U. Gd_4_ ^3+^(AlPCS_4_)_3_ ^4–^ nanoagent generating ^1^O_2_ for photodynamic therapy. Adv Funct Mater. (2018) 28:1801074. doi: 10.1002/adfm.201801074

[137] Shi HX Sun WC Wang Q Gu GY Si WL Huang W. A thienyl–substituted diketopyrrolopyrrole derivative with efficient reactive oxygen species generation for photodynamic therapy. ChemPlusChem. (2016) 81:515–20. doi: 10.1002/cplu.201600101 31968917

[138] Miolo G Sturaro G Cigolini G Menilli L Tasso A Zago I. 4, 6, 4’–trimethylangelicin shows high anti-proliferative activity on DU 145 cells under both UVA and blue light. Cell Proliferat. (2018) 51:e12430. doi: 10.1111/cpr.12430 PMC6528856 29318693

[139] Chakrabortty S Agrawalla BK Stumper A Vegi NM Fischer S Reichardt C. Mitochondria targeted protein–ruthenium photosensitizer for efficient photodynamic applications. J Am Chem Soc. (2017) 139:2512–9. doi: 10.1021/jacs.6b13399 PMC5588099 28097863

[140] Alves CG Lima–Sousa R de Melo–Diogo D Louro RO Correia IJ. IR780 based nanomaterials for cancer imaging and photothermal, photodynamic and combinatorial therapies. Int J Pharmaceut. (2018) 542:164–75. doi: 10.1016/j.ijpharm.2018.03.020 29549013

[141] Han HJ Zhang SM Wang Y Chen TT Jin Q Chen YJ. Biomimetic drug nanocarriers prepared by mini emulsion polymerization for near–infrared imaging and photothermal therapy. Polymer. (2016) 82:255–61. doi: 10.1016/j.polymer.2015.11.022

[142] Rejinold NS Choi G Choy JH. Recent developments on semiconducting polymer nanoparticles as smart photo–therapeutic agents for cancer treatments–a review. Polymers. (2021) 13:981. doi: 10.3390/polym13060981 33806912 PMC8004612

[143] Chang KW Gao DY Qi QF Liu YB Yuan Z. Engineering biocompatible benzodithiophene–based polymer dots with tunable absorptions as high–efficiency theranostic agents for multiscale photoacoustic imaging–guided photothermal therapy. Biomater Sci–UK. (2019) 7:1486–92. doi: 10.1039/c8bm01577e 30672925

[144] Sukwong P Somkid K Kongseng S Pissuwan D Yoovathaworn K. Respiratory tract toxicity of titanium dioxide nanoparticles and multi–walled carbon nanotubes on mice after intranasal exposure. Micro Nano Lett. (2016) 11:183–7. doi: 10.1049/mnl.2015.0523

[145] Luo Z Li ZQ Xie Z Sokolova IM Song L Peijnenburg WJGM. Rethinking nano–TiO_2_ safety: overview of toxic effects in humans and aquatic animals. Small. (2020) 16:2002019. doi: 10.1002/smll.202002019 32761797

[146] Kose O Tomatis M Leclerc L Belblidia NB Hochepied JF Turci F. Impact of the physicochemical features of TiO_2_ nanoparticles on their *in vitro* toxicity. Chem Res Toxicol. (2020) 33:2324–37. doi: 10.1021/acs.chemrestox.0c00106 32786542

[147] Geppert M Schwarz A Stangassinger LM Wenger S Wienerroither LM Ess S. Interactions of TiO_2_ nanoparticles with ingredients from modern lifestyle products and their effects on human skin cells. Chem Res Toxicol. (2020) 33:1215–25. doi: 10.1021/acs.chemrestox.9b00428 PMC7238409 32088960

[148] Zhang LW Monteiro–Riviere NA. Toxicity assessment of six titanium dioxide nanoparticles in human epidermal keratinocytes. Cutan Ocul Toxicol. (2019) 38:66–80. doi: 10.1080/15569527.2018.1527848 30265130

[149] Jafari S Mahyad B Hashemzadeh H Janfaza S Gholikhani T Tayebi L. Biomedical applications of TiO_2_ nanostructures: recent advances. Int J Nanomed. (2020) 15:3447–70. doi: 10.2147/IJN.S249441 PMC7234979 32523343

[150] De Angelis I Barone F Zijno A Bizzarri L Russo MT Pozzi R. Comparative study of ZnO and TiO_2_ nanoparticles: physicochemical characterization and toxicological effects on human colon carcinoma cells. Nanotoxicology. (2013) 7:1361–72. doi: 10.3109/17435390.2012.741724 23078188

[151] Çeşmeli S Biray Avci C. Application of titanium dioxide (TiO_2_) nanoparticles in cancer therapies. J Drug Targeting. (2019) 27:762–6. doi: 10.1080/1061186X.2018.1527338 30252540

[152] Rashid MM Forte Tavčer P Tomšič B. Influence of titanium dioxide nanoparticles on human health and the environment. Nanomaterials. (2021) 11:2354. doi: 10.3390/nano11092354 34578667 PMC8465434

[153] Shi JP Li J Wang Y Zhang CY. TiO_2_–based nanosystem for cancer therapy and antimicrobial treatment: a review. Chem Eng J. (2022) 431:133714. doi: 10.1016/j.cej.2021.133714

[154] Hou ZY Zhang YX Deng KR Chen YY Li XJ Deng XR. UV–emitting upconversion–based TiO_2_ photosensitizing nanoplatform: near–infrared light mediated *in vivo* photodynamic therapy via mitochondria–involved apoptosis pathway. ACS Nano. (2015) 9:2584–99. doi: 10.1021/nn506107c 25692960

[155] Chen ZF Zhao F Wang X Gao YY Li JX Chen LL. Organs distribution and injury after repeated intratracheal instillations of nano–In_2_O_3_ particles into the lungs of wistar rats. J Nanosci Nanotechno. (2020) 20:1383–90. doi: 10.1166/jnn.2020.17173 31492298

[156] Alaizeri ZM Alhadlaq HA Aldawood S Akhtar MJ Aziz AA Ahamed M. Photocatalytic degradation of methylene blue and anticancer response of In_2_O_3_/RGO nanocomposites prepared by a microwave–assisted hydrothermal synthesis process. Molecules. (2023) 28:5153. doi: 10.3390/molecules28135153 37446815 PMC10343526

[157] Pieta IS Gieroba B Kalisz G Pieta P Nowakowski R Naushad M. Developing benign Ni/g–C_3_N_4_ catalysts for CO_2_ hydrogenation: activity and toxicity study. Ind Eng Chem Res. (2022) 61:10496–510. doi: 10.1021/acs.iecr.2c00452 PMC9344432 35938051

[158] Taheri H Unal MA Sevim M Gurcan C Ekim O Ceylan A. Photocatalytically active graphitic carbon nitride as an effective and safe 2D material for *in vitro* and *in vivo* photodynamic therapy. Small. (2020) 16:1904619. doi: 10.1002/smll.201904619 31971659

[159] Ghazal S Mirzaee M Darroudi M. Green synthesis of tungsten oxide (WO_3_) nanosheets and investigation of their photocatalytic and cytotoxicity effects. Micro Nano Lett. (2022) 17:286–98. doi: 10.1049/mna2.12134

[160] Mano SS Kanehira K Sonezaki S Taniguchi A. Effect of polyethylene glycol modification of TiO_2_ nanoparticles on cytotoxicity and gene expressions in human cell lines. Int J Mol Sci. (2012) 13:3703–17. doi: 10.3390/ijms13033703 PMC3317737 22489177

[161] Park MVDZ Neigh AM Vermeulen JP de la Fonteyne LJJ Verharen HW Briedé JJ. The effect of particle size on the cytotoxicity, inflammation, developmental toxicity and genotoxicity of silver nanoparticles. Biomaterials. (2011) 32:9810–7. doi: 10.1016/j.biomaterials.2011.08.085 21944826

[162] Karlsson J Vaughan HJ Green JJ. Biodegradable polymeric nanoparticles for therapeutic cancer treatments. Annu Rev Chem Biomol. (2018) 9:105–27. doi: 10.1146/annurev-chembioeng-060817-084055 PMC6215694 29579402

[163] Wang XY Mu YZ Yang KK Shao K Cong X Cao Z. Reversible regulation of the reactive oxygen species level using a semiconductor heterojunction. ACS Appl Mater Interfaces. (2022) 14:46324–39. doi: 10.1021/acsami.2c13956 36200707

[164] Tavakoli S Kharaziha M Nemati S. Polydopamine coated ZnO rod–shaped nanoparticles with noticeable biocompatibility, hemostatic and antibacterial activity. Nano-Struct Nano-Objects. (2021) 25:100639. doi: 10.1016/j.nanoso.2020.100639

[165] Mishra YK Adelung R. ZnO tetrapod materials for functional applications. Mater Today. (2018) 21:631–51. doi: 10.1016/j.mattod.2017.11.003

[166] Wahab R Khan F Mishra YK Musarrat J Al–Khedhairy AA. Antibacterial studies and statistical design set data of quasi zinc oxide nanostructures. RSC Adv. (2016) 6:32328–39. doi: 10.1039/c6ra05297e

[167] Wahab R Khan F Al–Khedhairy AA. Peanut–shaped ZnO nanostructures: A driving force for enriched antibacterial activity and their statistical analysis. Ceram Int. (2020) 46:307–16. doi: 10.1016/j.ceramint.2019.08.264

[168] AbuMousa RA Baig U Gondal MA AlSalhi MS Alqahtani FY Akhtar S. Photo–catalytic killing of HeLa cancer cells using facile synthesized pure and Ag loaded WO_3_ nanoparticles. Sci Rep–UK. (2018) 8:15224. doi: 10.1038/s41598-018-33434-7 PMC6189059 30323306

[169] Akhtar MJ Ahamed M Kumar S Khan MM Ahmad J Alrokayan SA. Zinc oxide nanoparticles selectively induce apoptosis in human cancer cells through reactive oxygen species. Int J Nanomed. (2012) 7:845–57. doi: 10.2147/IJN.S29129 PMC3289443 22393286

[170] Xia T Kovochich M Liong M Madler L Gilbert B Shi H. Comparison of the mechanism of toxicity of zinc oxide and cerium oxide nanoparticles based on dissolution and oxidative stress properties. ACS Nano. (2008) 2:2121–34. doi: 10.1021/nn800511k PMC3959800 19206459

[171] Zhou ZJ Song JB Nie LM Chen XY. Reactive oxygen species generating systems meeting challenges of photodynamic cancer therapy. Chem Soc Rev. (2016) 45:6597–626. doi: 10.1039/c6cs00271d PMC5118097 27722328

[172] Yi HX Cheng ZN. A literature review on high–performance photocatalysts for sustainable cancer therapy. Crystals. (2021) 11:1241. doi: 10.3390/cryst11101241

[173] Jafari M Rezvanpour A. Upconversion nano–particles from synthesis to cancer treatment: a review. Adv Powder Technol. (2019) 30:1731–53. doi: 10.1016/j.apt.2019.05.027

[174] Gu B Pliss A Kuzmin AN Baev A Ohulchanskyy TY Damasco J. In–situ second harmonic generation by cancer cell targeting ZnO nanocrystals to effect photodynamic action in subcellular space. Biomaterials. (2016) 104:78–86. doi: 10.1016/j.biomaterials.2016.07.012 27442221

[175] Wang C Cheng L Liu Z. Upconversion nanoparticles for photodynamic therapy and other cancer therapeutics. Theranostics. (2013) 3:317–30. doi: 10.7150/thno.5284 PMC3645058 23650479

[176] Li K Hong E Wang B Wang ZY Zhang LW Hu RX. Advances in the application of upconversion nanoparticles for detecting and treating cancers. Photodiagn Photodyn. (2019) 25:177–92. doi: 10.1016/j.pdpdt.2018.12.007 30579991

[177] Zhang P Steelant W Kumar M Scholfield M. Versatile photosensitizers for photodynamic therapy at infrared excitation. J Am Chem Soc. (2007) 129:4526–7. doi: 10.1021/ja0700707 PMC2528873 17385866

[178] Wang WN Zhang F Zhang CL Guo YC Dai W Qian HS. Fabrication of zinc oxide composite microfibers for Near–Infrared–Light–Mediated photocatalysis. ChemCatChem. (2017) 9:3611–7. doi: 10.1002/cctc.201700781

[179] Nyangaresi PO Qin Y Chen GL Zhang BP Lu YH Shen L. Comparison of UV–LED photolytic and UV–LED/TiO_2_ photocatalytic disinfection for Escherichia coli in water. Catal Today. (2019) 335:200–7. doi: 10.1016/j.cattod.2018.11.015

[180] Wei B Dong F Yang W Luo CH Dong QJ Zhou ZQ. Synthesis of carbon–dots@SiO_2_@TiO_2_nanoplatform for photothermal imaging induced multimodal synergistic antitumor. J Adv Res. (2020) 23:13–23. doi: 10.1016/j.jare.2020.01.011 32071788 PMC7016282

[181] Li W Zhao DY. Extension of the Stöber method to construct mesoporous SiO_2_ and TiO_2_ shells for uniform multifunctional core–shell structures. Adv Mater. (2013) 25:142–9. doi: 10.1002/adma.20120354 23397611

[182] Mungondori HH Tichagwa L Green E. Synthesis and glass immobilization of carbon and nitrogen doped TiO_2_–SiO_2_ and its effect on E. coli ATCC 25922 bacteria. Briti J Appl Sci Technol. (2014) 5:447–60. doi: 10.9734/BJAST/2015/11049

[183] Kang Y Lei L Zhu CF Zhang HJ Mei L Ji XY. Piezo–photocatalytic effect mediating reactive oxygen species burst for cancer catalytic therapy. Mater Horiz. (2021) 8:2273–85. doi: 10.1039/d1mh00492a 34846431

[184] Zheng HJ Lin HM Tian H Lin KL Yang F Zhang XH. Steering piezocatalytic therapy for optimized tumoricidal effect. Adv Funct Mater. (2024) 34:2400174. doi: 10.1002/adfm.202400174

[185] Yu Q Shi WH Li S Liu H Zhang JM. Vancements in piezoelectric nanomaterials for dynamic tumor therapy. Molecules. (2023) 28:3170. doi: 10.3390/molecules28073170 37049933 PMC10095813

[186] Jia PW Li JM Huang HW. Piezocatalysts and piezo–photocatalysts: from material design to diverse applications. Adv Funct Mater. (2024), 2407309. doi: 10.1002/adfm.202407309

[187] Cheng SS Luo Y Zhang J Shi R Wei ST Dong KJ. The highly effective therapy of ovarian cancer by Bismuth–doped oxygen–deficient BaTiO_3_ with enhanced sono–piezocatalytic effects. Chem Eng J. (2022) 442:136380. doi: 10.1016/j.cej.2022.136380

[188] Fooladi S Nematollahi MH Iravani S. Nanophotocatalysts in biomedicine: Cancer therapeutic, tissue engineering, biosensing, and drug delivery applications. Environ Res. (2023) 231:116287. doi: 10.1016/j.envres.2023.116287 37263475

[189] Meng JX Zhang PC Zhang FL Liu HL Fan JB Liu XL. A self–cleaning TiO_2_ nanosisal–like coating toward disposing nanobiochips of cancer detection. ACS Nano. (2015) 9:9284–91. doi: 10.1021/acsnano.5b04230 26285086

